# Rutin, A Natural Inhibitor of IGPD Protein, Partially Inhibits Biofilm Formation in *Staphylococcus xylosus* ATCC700404 *in vitro* and *in vivo*

**DOI:** 10.3389/fphar.2021.728354

**Published:** 2021-08-11

**Authors:** Qianwei Qu, Wenqiang Cui, Xiaoxu Xing, Rongfeng Zou, Xingyu Huang, Xiaozhen Wang, Tong Wu, God’spower Bello-Onaghise, Shuguang Yuan, Yanhua Li

**Affiliations:** ^1^ Heilongjiang Key Laboratory for Animal Disease Control and Pharmaceutical Development, College of Veterinary Medicine, Northeast Agricultural University, Harbin, China; ^2^ Shenzhen Institutes of Advanced Technology, University of Chinese Academy of Sciences, Beijing, China; ^3^ Institute of Biomedicine and Biotechnology, Shenzhen Institutes of Advanced Technology, Chinese Academy of Sciences, Shenzhen, China; ^4^ College of Animal Science and Technology, Heilongjiang Bayi Agricultural University, Daqing, China; ^5^ Department of Animal Science, Faculty of Agriculture, University of Benin, Benin, Nigeria

**Keywords:** *S. xylosus*, biofilm, rutin, IGPD, virulence factors, enzyme inhibitor

## Abstract

*Staphylococcus xylosus* (*S. xylosus*) has become an emerging opportunistic pathogen due to its strong biofilm formation ability. Simultaneously, the biofilm of bacteria plays an important role in antibiotic resistance and chronic infection. Here, we confirmed that rutin can effectively inhibit biofilm formation in *S. xylosus*, of which the inhibition mechanism involves its ability to interact with imidazole glycerol phosphate dehydratase (IGPD), a key enzyme in the process of biofilm formation. We designed experiments to target IGPD and inhibited its activities against *S. xylosus*. Our results indicated that the activity of IGPD and the amount of histidine decreased significantly under the condition of 0.8 mg/ml rutin. Moreover, the expression of IGPD mRNA (*hisB*) and IGPD protein was significantly down-regulated. Meanwhile, the results from molecular dynamic simulation and Bio-layer interferometry (BLI) technique showed that rutin could bind to IGPD strongly. Additionally, *in vivo* studies demonstrated that rutin treatment reduced inflammation and protect mice from acute mastitis caused by *S. xylosus*. In summary, our findings provide new insights into the treatment of biofilm mediated persistent infections and chronic bacterial infections. It could be helpful to design next generation antibiotics to against resistant bacteria.

## Introduction

*Staphylococcus xylosus* (*S. xylosus*) is a coagulase-negative, Gram-positive coccoid organism that was first identified in 1975 (Schleifer and Kloos, 1975). It is a common bacterium in the environment and has been linked to opportunistic infections in both humans and animals. Although there are few reports on the pathogenicity of *S. xylosus*, some cases of *S. xylosus* infection have been reported in recent years, including erythema nodosum Giordano et al. (2016), pyelonephritis Tselenis-Kotsowilis et al. (1982), mastitis Bochniarz et al. (2014) and corneal infections (Makki et al., 2011). In addition, *S. xylosus* was also identified as one of the most abundant *staphylococcus* bacterial isolates on the post-male circumcision coronal sulcus (Liu et al., 2013). This has drawn the attention of researchers and clinicians to the potential public health threat of *S. xylosus*.

What is more serious is that *S. xylosus* has a strong ability to form biofilm (Tremblay et al., 2014; Xu et al., 2017). Biofilm was considered as a key virulence factor that may offer protection against different antimicrobial strategies to develop recurrent infections under physiological, metabolic and/or immunological stress environments (Breser et al., 2018; Conrad and Poling-Skutvik, 2018). Meanwhile, the biofilm formation comprises of a number of well-organized steps including adhere to a surface, formation of microcolonies, formation of young biofilm, and subsequent dispersal of mature biofilm (Yang et al., 2012). Adhesion stage plays a key role in the formation of biofilm. Bacteria gather on adherent surfaces by cilia or flagella. With the increase of aggregates, the related components of bacterial biofilm, such as polysaccharides, lipids, proteins and DNA, are gradually discharged from the body to form extracellular matrix, which encapsulates the bacteria, and then maintains the structure and function of bacterial biofilm (Otero et al., 2013). Thus, it is urgent and critical to find novel antibiotics with the ability to interfere with the process biofilm formation in bacteria*.* This will help mitigate chronic and persistent infections mediated by biofilm formed by bacteria such as *S. xylosus.*

L-histidine synthesis, as one of the pathways of nitrogen metabolism pathway, is related to biofilm formation. When the nitrogen-deficient medium transitions to an L-histidine-rich environment, the biofilm formation ability of *Saccharomyces cerevisiae* is significantly reduced. The mechanism of action is the result of the interaction between L-histidine and glycosylated mucin on the cell wall. On the contrary, adding L-histidine to the medium containing the nitrogen source will promote the co-aggregation of *Azotobacter chroococcum* and enhance its biofilm formation ability (Zeidan et al., 2014; Velmourougane and Prasanna, 2017). Furthermore, imidazole glycerol phosphate dehydratase (IGPD) is a major enzyme in the pathway of histidine biosynthesis in bacteria and plants (Alifano et al., 1996; Rawson et al., 2018). IGPD catalyzes the sixth step of histidine biosynthesis which involves a dehydration reaction to produce imidazole acetol phosphate (IAP) from imidazole glycerol phosphate (IGP) and a concomitant water molecule (Carsiotis and Jones, 1974; Chiariotti et al., 1986; Gohda et al., 1998; Bisson et al., 2015). IGPD is often used as a target for herbicides because of its unique biological characteristics (Hilton et al., 1965; Rawson et al., 2018). The presence of two molecules of the metal ion (Mn^2+^) at the active center of IGPD plays an important role in catalyzing substrate reaction (Hilton et al., 1965). In a previous study, we have demonstrated that IGPD plays an important regulatory role in the biofilm formation of *S. xylosus* (Zhou et al., 2018). Therefore, IGPD has been considered a potential target in solving the menace of *S. xylosus.* Meanwhile, the spatial structure of IGPD was predicted by homologous modeling, and it was used as the basis for screening the enzyme inhibitor (Chen et al., 2017).

Rutin, also known as vitamin *p*, is a major flavonoid. It has been reported to possess antiplatelet, antiviral and antihypertensive properties. It also enhances the functions of blood capillaries because of its high cardioprotective and antioxidant activity (Korkmaz and Kolankaya, 2010). In addition, the antibiofilm activity of rutin has also been reported. For example, data obtained from Metabolomics-based screening revealed that burdock leaf significantly inhibited the formation of biofilm by *Pseudomonas aeruginosa* due to its high rutin content (Lou et al., 2015). This was further corroborated by findings from other authors. It was observed that rutin obtained from aqueous extract of *syringa oblata lindl* inhibited the biofilm formation by *S. suis* Bai et al. (2017), *S. xylosus* Chen et al. (2019), *E. coli* and *S. aureus* (Al-Shabib et al., 2017). Although the effect of rutin on the biofilm of *S. xylosus* has been investigated Al-Shabib et al. (2017), Chen et al. (2019), Liu et al. (2019), however, the atomic mechanisms involved in this progression have not been fully elucidated.

In addition, some recent reports have also described that rutin was a protease inhibitor (Al-Harbi et al., 2019). Ragunathan and Ravi have used molecular docking to suggest that rutin may be a potential inhibitor of protein (Ragunathan and Ravi, 2015). So far, there is no report on the inhibitory effect of rutin on the proteins against biofilm formation. In this study, we systematically evaluated the mechanism by which rutin disrupts the formation of biofilm in *S. xylosus*. We predicted the potential binding mode between rutin and IGPD by computer calculations in combination of mutagenesis. Furthermore, a mouse mastitis model was used to verify the anti-virulence factor properties of rutin *in vivo*. The pathogenicity of *S. xylosus* was significantly mitigated and all symptoms of bacteremia corrected after rutin intervention.

## Materials and Methods

### Bacterial Strain and Growth Conditions

The bacterial species and plasmids used in this study are listed in Supplementary Table S1 *. S. xylosus* was grown at 37°C in Tryptic Soy Broth (TSB). The cultures were used for the biofilm assays and other bacterial experiments. *E. coli* was grown in LB medium at 37°C. The cultures were used to amplify plasmids and express proteins. Dissolve 8 mg/ml rutin in 10% DMSO as a backup solution.

### Determination of Minimum Inhibitory Concentration of Rutin

The effect of the minimum inhibitory concentration of rutin on the growth of *S. xylosus* was repeated three times, but no concentration of rutin significantly inhibited the growth of the bacteria. The growth curve of bacteria was drawn to determine whether rutin had an effect on the growth of *S. xylosus*. Overnight culture of bacterial strains was grown at 37°C for 24 h. Then TSB medium was used to dilute the bacterial cultures to 1×10^5^ colony forming units/mL. This was followed by the addition of 0.8 mg/ml rutin. The mixture was grown in an incubator for 12 h at 37°C and the absorbance was read with a UV spectrophotometer (Thermo Scientific™ Evolution 201, NY, United States) every 1 h at 600 nm wavelength. Finally, the growth curve of bacteria was drawn using the values obtained from the UV spectrophotometer.

### Determining the Interference of Rutin on Biofilm Formation

The procedure was done in line with an earlier procedure described by Zhou (Zhou et al., 2018). *S. xylosus* and *ΔhisB S. xylosus* were cultured at the mid-exponential growth phase to an optical density of 0.1 at OD_590_. Cultures and different concentrations of rutin (0.8 mg/ml,0.4 mg/ml and 0.2 mg/ml) were added to each well of a 96-well microplate or a 6-well microplate (Corning Costar® 3599 Corning, NY, United States). In addition, a negative control group (bacterial culture without rutin) was setup. After incubated at 37°C for 6, 12 and 24 h, crystal violet staining was performed and followed by scanning electron microscopy analysis. The biofilms were obtained from bacterial cells and prepared for analysis as described by Zhou (Zhou et al., 2018).

### Molecular Docking of Rutin and IGPD

Homology modeling of *S. xylosus* IGPD and model validation have been described in our previous study (Chen et al., 2017). The 3D structures of rutin were downloaded from Pubchem Database (https://pubchem.ncbi.nlm.nih.gov/). The Ligand model obtained from the DS interface was used to optimize rutin to generate different conformers for docking analysis in Discovery Studio 3.0 (DS 3.0). We defined the two Mn^2+^ in IGPD as the potential active pocket. Hence, other parameters were set to default. The best binding mode obtained was analyzed and visualized by PyMOL v2.3 software.

### Molecular Dynamics (MD) Simulation

The long-time-scale MD simulations were performed in Desmond Bowers et al. (2006) for the docked IGPD/rutin complex. The Desmond force field parameters for among IGPD, rutin, and Mn^2+^, were described in previous article (Fu et al., 2018). We used 23 Na^+^ and 44 Cl^−^ ions to make the system neutral and to set the ionic strength to 0.15 M. The total number of atoms in the investigated system was approximately 50,000 including about 8,300 water molecules. The periodic box dimensions were set to 7.0 nm × 7.0 nm × 10.4 nm. The results obtained from the MD simulations were analyzed and visualized in Maestro.

### Expression and Purification of IGPD and Its Site Directed Mutant Protein

Purified IGPD protein was obtained from our previous studies. At the same time, the pET30a-*hisB* mutant was made by introducing a point mutation (Arg7, His62, His63, Glu66, Asp97, Arg110, His159, Glu162 and Lys166 to Ala) via overlap PCR and subsequently verified by sequencing (BGI). The vectors were subsequently cloned into a pET30a vector for protein expression. For IGPD and pET30a-*hisB* mutant, insoluble material was removed by centrifugation and cell-free extracts were purified in a process involving affinity chromatography and molecular sieve chromatography.

### Interactions of rutin with IGPD and its site directed mutant protein by Bio-layer interferometry (BLI)

BLI experiments were performed using an Octet system (Forte Bio) placed in PBS pH 7.4, 0.05% (v/v) Tween-20 and 1 mg/ml BSA running buffer at room temperature (25°C). Freshly prepared IGPD protein (50 μg/ml) was coupled to the tip of a Forte Bio Octet NTA instrument. A dilution series of rutin (500,000 nM to 7,813 nM) was used to measure a dose-response curve of association and dissociation. The dissociation period was set at 60 s. The brief experimental process is shown in Supplementary Figure S1.

### Effect of Rutin on IGPD Activity

*S. xylosus* was grown in TSB for 6, 12 and 24 h respectively with 0.8 mg/ml rutin, no rutin was added to the control groups. The bacterial culture was centrifuged at 13,200 g for 5 min. The supernatant was collected and centrifuged at 600 × g for 5 min, then the cells were sonicated on ice for 15 min. The activity of the enzyme was determined using a previously described stopped-assay protocol Wroe et al. (2020) with minor modifications. The reaction mixture consisted of PBS buffer pH 7.4, and IGPD. The reaction was carried out at 37°C using IGP (Santa Cruz Biotechnology, United States). The reaction was stopped by adding sodium hydroxide at the point with an interval of 30 s. The mixture was then incubated at 37°C for 20 min to convert the product imidazole acetol-phosphate (IAP) into an enolized form, the absorbance was read at 280 nm using a Shimadzu UV spectrophotometer against a blank. The extinction coefficient of IAP formed under these conditions is as reported previously (Martin and Goldberger, 1967).

### Real Time RT-PCR

We investigated the effect of the 0.8 mg/ml rutin on the gene expression of *hisB* in *S. xylosus*. Incubation was conducted at 37°C for 6, 12 and 24 h respectively. Briefly, bacteria strains were collected by centrifugation (10,000 g for 5 min) and treated with an RNASE REMOVER (Huayueyang Ltd., Beijing, China). Total RNA was extracted with a bacterial RNA isolating kit (Omega, Beijing, China) and then, reverse transcription of total RNA into cDNA was carried out using a reverse transcription Kit. Relative copy numbers and expression ratios of the selected genes were normalized to the expression of house-keeping gene (*16S-rRNA* gene) (Woese, 1987; Attaran et al., 2017). The specific primers (*hisB* gene: *Forward*: *TAC​TTC​TGT​ATC​ACC​ATT*, *Reverse*: *ACT​ATC​TAT​CTC​ACT​TGC*. *16S-rRNA* gene: *Forward*: *CGG​GCA​ATT​TGT​TTA​GCA*, Reverse: *ATT​AGG​TGG​AGC​AGG​TCA*) used for the quantitative RT-PCR (Takara Biomedical Technology Co., Ltd. Beijing, China) were purchased from Takara. The quantitative RT-PCR procedure was performed as described by (Zhou et al., 2018). In addition, gene expression was calculated by relative quantitative method according to the following formula.

$$2^{-(\Delta Ct\left(After\;dosing\right)-\Delta Ct\left(Before\;dosing\right))}$$

### Western Blot

Anti-IGPD polyclonal antibody was obtained from our previous studies (Qu et al., 2019). The effect of 0.8 mg/ml rutin on the expression of IGPD in *S. xylosus* was also investigated. Bacteria were cultured in 0.8 mg/ml rutin solution at 37°C for 6, 12 and 24 h respectively. Cells without rutin served as control. Appropriate amount of PBS buffer solution was added to the harvested bacterial strains, and the solution was subjected to ultrasonic screening. This was followed by the centrifugation (10,000 g for 5 min) of the supernatants to obtain protein samples. Finally, western blot analysis was performed according to the standard procedure (Yang et al., 2007).

### Determination of Histidine Content

*S. xylosus* and the *ΔhisB S. xylosus* were grown in 0.8 mg/ml rutin at 37°C for 6, 12, and 24 h, respectively. The pellets were then suspended in sterile double distilled water and the bacterial culture was sonicated to release histidine. The mixture was filtered using a 0.45 μm filter, and the histidine content in the bacteria was determined by high performance liquid chromatography (HPLC) on a Waters Alliance HPLC system (Waters e2695, United States). Quantification of the histidine in the sonicated treated bacteria was done in HPLC at 205 nm against concentration using the external standard method. The specific details of the complex system have been previously described (Qu et al., 2019).

### Animal Experiment

This experiment was conducted in line with the procedure already established in our laboratory. All animal experiments were conducted in accordance with the U.S. National Institutes of Health (NIH) Guide for the Care and Use of Laboratory Animals (NIH Publication No. 80-23, revised in 1996). All animal experiments involved in this experiment were approved by the experimental animal research ethics committee of Northeast Agricultural University (SRM-11). Lactating BALB/c mice obtained from the Experimental Animal Center of the Second Affiliated Hospital of Harbin Medical University were housed in a controlled environment (specific pathogen-free conditions). Briefly, aliquot obtained from overnight bacterial culture was inoculated into fresh TSB broth (1:100) and incubated until the OD_600_ reached 0.8. Cells were harvested by centrifugation (10,000 g for 5 min), washed three times with PBS and diluted to a bacterial count of 10^9^ CFU/ml for mice mammary gland infection. In order to establish a mastitis model of infection, lactating BALB/c mice, 10–12 weeks of age and weighing 30–32 g, were given sodium pentobarbital as anesthesia. They were then infected by injecting the canal glands with 50 μl/kg of body weight of *S. xylosus* suspension at the 4th breast (L4 and R4) on both sides of the lower abdomen counting from the head. Before the infection, the same method was used to administer 50 μL of rutin (2 mg/kg) solution. The normal (uninfected) mice served as an untreated control. All the mice were sacrificed through the inhalation of CO_2_ at 48 h post-infection. Hereafter, tissues from the mammary gland were isolated and fixed in 4% paraformaldehyde, staining was done with hematoxylin and eosin, and visualization was done using light microscopy. The expression levels of the inflammatory cytokines, TNF-α and IL-6 in the supernatants were also quantified using ELISA kits.

### Statistical Analysis

Assays were done three times. Data were subjected to statistical analysis and the *t* test and two-way ANOVA test were conducted by GraphPad Prism 8 software. The values were reported as mean ± standard deviation (SD). *p* < 0.05 was considered to be statistically significant.

## Results and Discussion

### Determination of the Ability of Rutin to Inhibit the Biofilm Formation of *S. xylosus*

**FIGURE 1:**
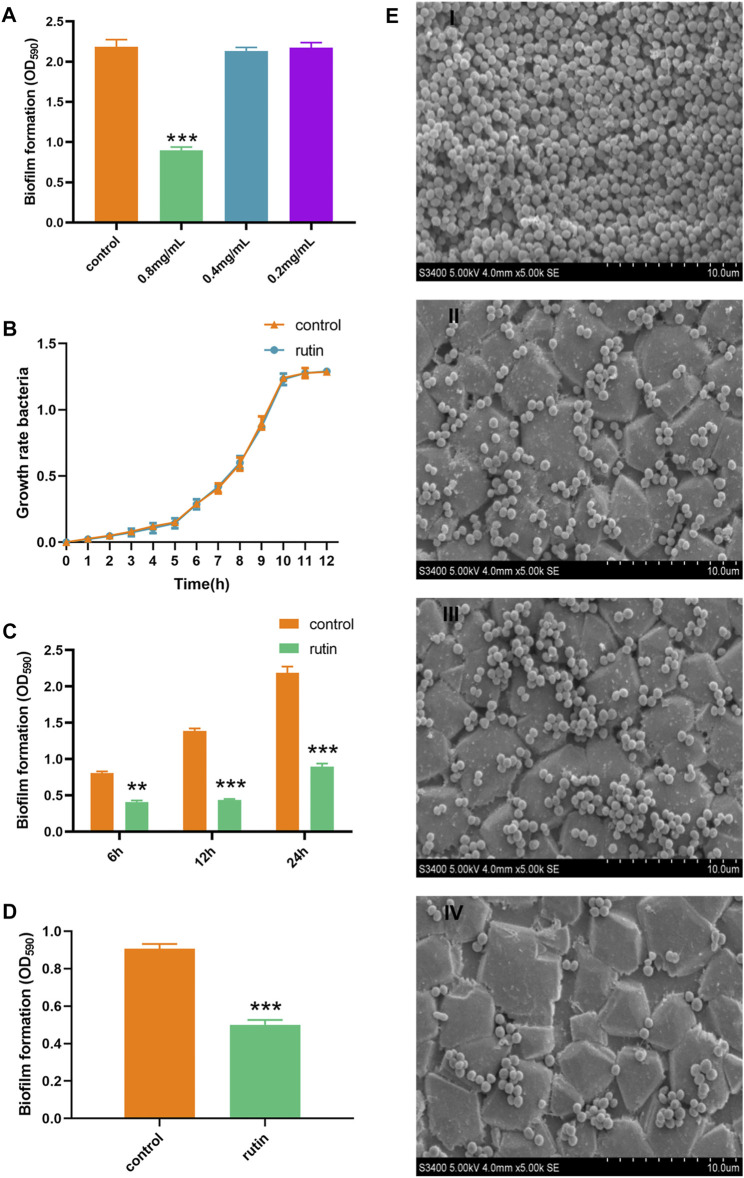
Interference effect of Rutin on the biofilm formation of *S. xylosus* ATCC700404. **(A):** Interference effect of rutin with different concentrations on the biofilm formation of *S. xylosus*, 0.8 mg/ml rutin significantly inhibited the formation of biofilm, but the other two concentrations did not. **(B):** The effect of rutin on the growth rate of *S. xylosus.* **(C):** The effect of 0.8 mg/ml rutin on the biofilm of *S. xylosus* at different growth stages. When the bacteria were incubated for 6, 12, and 24 h, respectively, rutin significantly inhibited the formation of the biofilm. **(D):** Rutin inhibited the formation of the biofilm of *ΔhisB S. xylosus* at 24 h. **E:** The effect of rutin on the biofilm morphology of *S. xylosus* was observed by SEM. Scanning electron micrographs of *S. xylosus* biofilm following growth in TSB supplemented with 0.8 mg/ml of rutin (II), or control (I). Controls refer to the cultures without rutin. Scanning electron micrographs of *ΔhisB S. xylosus* biofilm following growth in TSB supplemented with 0.8 mg/ml of rutin (IV), or control (III). Controls refer to the cultures without rutin. The asterisk indicates statistical significance by *t* test and two-way ANOVA test with *p* < *0.05*.

In this study, it was observed that 0.8 mg/ml rutin could effectively interfere with the formation of biofilm by *S. xylosus* even as early as within 6 h of intervention (Figures 1A, C and E). This may be due to rutin inhibiting bacterial adhesion. At the same time, in order to exclude the decrease of biofilm formation ability caused by the decrease of bacteria concentration not caused by rutin, we measured the growth curve. The results indicated that, 0.8 mg/ml rutin had no effect on the growth rate of *S. xylosus* during a 0–12 h incubation at 37°C (Figure 1B). According to earlier reports, rutin could interfere with the adhesion phase of *Streptococcus suis* and then abrogate the formation of its biofilm (Bai et al., 2017; Wang et al., 2017). In addition, the bacterial quorum sensing system is also closely related to the early adhesion of bacteria. At the same time, rutin could interrupt the production of AI-2 to inhibit quorum sensing system of *Escherichia coli*, reduce bacterial adhesion, and then interfere with the formation of biofilm in *E. coli* (Szabados et al., 2007). It was reported that in combination with gentamicin sulfate, rutin significantly weakened the adhesion of *Pseudomonas aeruginosa* and its ability to form biofilm (Lou et al., 2015). Therefore, the interference effect of rutin on *S. xylosus* biofilm at different time points (6, 12, and 24 h, respectively) may be related to the inhibition of bacterial adhesion. Interestingly, rutin also had an interference effect on the biofilm formation of *S. xylosus ΔhisB*, as showed in Figures 1D, E. This indicates that rutin is an extremely effective drug candidate to interfere with the biofilm formation of *S. xylosus*.

### Interaction Between IGPD and Rutin

**FIGURE 2:**
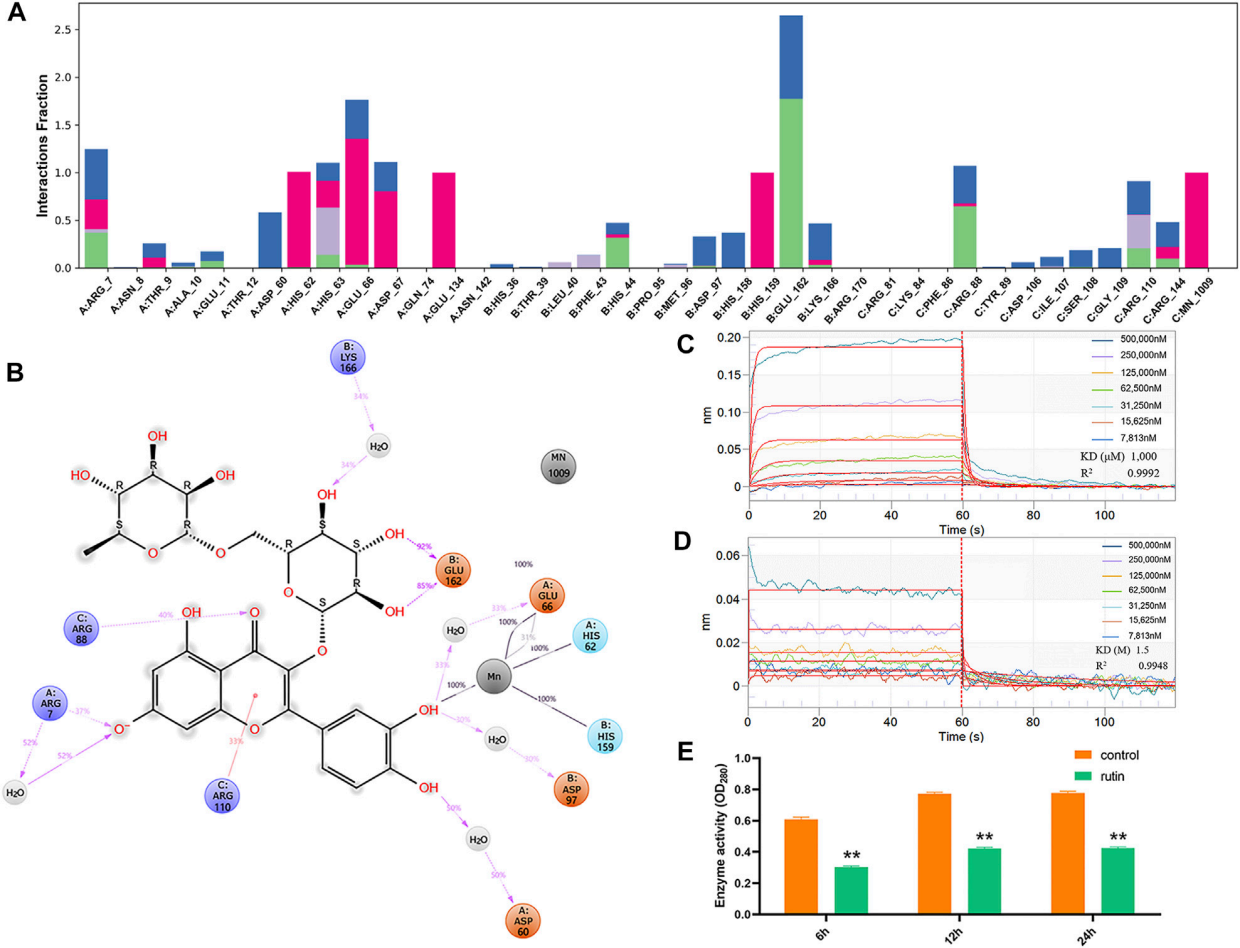
Verification of direct interaction between rutin and IGPD. **(A):** IGPD interactions with the rutin monitored throughout the simulation. **(B):** A schematic of detailed ligand atom interactions with the protein residues. **(C):** Kinetic analysis by BLI of the binding of rutin to IGPD. The Rutin binds to the IGPD with a high affinity (KD = 1,000 μM). **(D):** Kinetic analysis by BLI of the binding of rutin to ΔHis62. The rutin binds to the ΔHis62 with a low affinity (KD = 1.5 M). Response units were plotted against protein concentrations. The KD values were calculated by the ForteBio analysis software (ForteBio Data Analysis Version 11.0.2.3). **(E):** Determination of IGPD activity. The asterisk indicates statistical significance by two-way ANOVA test with *p* < *0.05*.

Previous studies have demonstrated that IGPD plays an important regulatory role in the formation of biofilm in *S. xylosus* (Zhou et al., 2018). Therefore, we carried out molecular interaction test and enzyme activity analysis test to verify this assumption. BLI analysis showed that rutin could interact with IGPD. When the rutin was 7,813 nM, a binding signal was observed on the surface of the sensor. In addition, when the concentration of rutin was between 7,813 nm and 500,000 nm, the interaction intensity showed a dose-dependent. When rutin was dissociated, the interaction disappeared (Figure 2C). Furthermore, we detected the binding ability of the supernatant to the substrate IGP, and analyzed the IGPD activity of *S. xylosus*. The bacteria strains were grown in media with or without (control) 0.8 mg/ml rutin for 6, 12, and 24 h, respectively. The reaction was significantly reduced in treated medium compared with the control (*p < 0.05*). The results showed that rutin inhibited the enzyme activity of IGPD (Figure 2E). Based on the above results, we speculate that rutin is a potential IGPD inhibitor, occupying the catalytic center of IGPD enzyme, preventing the binding of IGPD with its substrate, thus affecting the enzyme activity of *S. xylosus*.

Meanwhile, molecular docking was applied to followed by the MD simulation to illuminate the molecular mechanism between IGPD and rutin. The results shown that their binding mode was stable (Supplementary Figure S2), which was demonstrated by the small changes in RMSD values (2–3 Å) of rutin along with simulation time. Protein ligand interactions were monitored throughout the simulation. These interactions were categorized by type and summarized, in the plot above (Figure 2A). As showed in Figure 2B, His62, Glu66, and His159 of IGPD form coordination bonds with Mn^2+^, which helps stabilize the active binding pocket. We also found that rutin formed coordination bonds with the Mn^2+^, and formed H-bonds with Arg7, Arg88, Arg110, Glu162. In addition, the phenolic hydroxyl group of rutin also forms a very strong metal coordination bond with Mn2^+^. This may be the key to the interaction between rutin and IGPD.

### Identification of Rutin Binding Sites to IGPD

According to the results of MD simulation, we constructed and expressed nine mutated variance, the representative sequencing map, expression and purification results of the mutant protein are shown in supplementary Figures S3, S4 and S5. Meanwhile, we analyzed the interaction between rutin and mutant proteins (Supplementary Figures S6 A–H). As expected, the KD values of rutin and IGPD only changed about 2 times after the mutations of amino acids except for His62. And this change did not affect the interaction between rutin and IGPD, indicating that these eight amino acids are not the key amino acids in the interaction between rutin and IGPD. Interestingly, when His62 was mutated into Ala62 (ΔHis62), the KD value of rutin and IGPD decreased 1,500 times (Figure 2D), indicating that the affinity between ΔHis62 and rutin was very low, and there was almost no interaction between them. We know that His62 plays an important role in stabilizing Mn^2+^ (coordination bond) and protein small molecule interactions (hydrogen bond). Furthermore, IGPD is a kind of metal active enzyme, and its active site is around its Mn^2+^. As long as it prevents the substrate from contacting with Mn^2+^, it can inhibit the enzyme activity and make it unable to play its role (Petersen et al., 1997). In this study, the mutation of His62 resulted in the disappearance of the metal coordination bond between Ala62 and Mn^2+^. This made rutin to swing more freely in the active cavity, the instability of rutin in the active cavity is not suitable for the interaction between rutin and the surrounding amino acids, and cannot effectively occupy the active sites, thus making the inhibition of IGPD impossible. Furthermore, the mutation may result in a concomitant change in the distribution of the electron cloud of the amino acid residues in the active cavity to some extent, which may be unfavorable for the binding of rutin or change the structure of the active site, which may cause the radius of the active cavity to expand beyond normal (Grossmann et al., 1996).

### Regulation of Rutin on IGPD Expression

**FIGURE 3:**
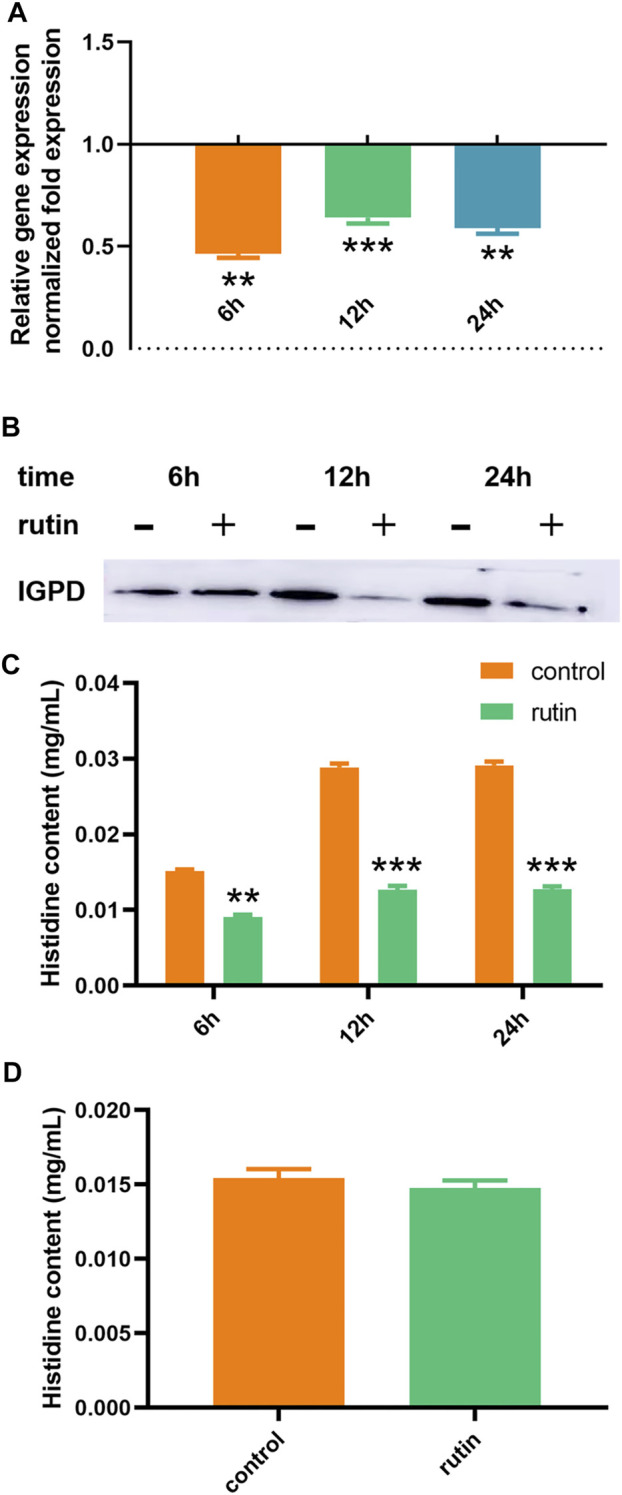
Regulation of rutin on IGPD of *S. xylosus*. **(A):** The effect of rutin on the expression of *hisB* gene in *S. xylosus* at the 6, 12 and 24 h, respectively. **(B):** The effect of rutin on the expression of IGPD protein in *S. xylosus* at 6, 12, and 24 h, respectively. **(C):** Rutin significantly reduced the histidine content of *S. xylosus* at 6, 12, and 24 h, respectively of bacterial growth. **(D):** The effect of rutin on the histidine content of the *ΔhisB S. xylosus* at the 24 h. The asterisk indicates statistical significance by two-way ANOVA test with *p* < *0.05*.

Our previous studies have shown that the deletion of the *hisB* gene will lead to a significant decrease in the biofilm formation ability of *S. xylosus*. Therefore, *hisB* plays an important role in the biofilm formation in *S. xylosus* (Zhou et al., 2018). In this study, we speculated that rutin may regulate the formation of biofilm in *S. xylosus* by regulating the transcription and translation levels of *hisB* gene leading to changes in the L-histidine content and enzyme activity. In order to verify this speculation, we measured the regulation of rutin on *hisB* mRNA expression at different time points by RT-PCR. The results showed that rutin significantly reduced the expression of *hisB* mRNA (Figure 3A). In order to further confirm this effect, we detected the effect of rutin on the expression of IGPD protein at different time points by Western blot. The result proved that rutin significantly reduced the expression of IGPD protein at 12 and 24 h, but did not affect the expression of IGPD protein at 6 h after treatment (Figure 3B). In addition, we observed that there was a significant difference between the gene expression and protein level at 6 h. The gene and protein expression levels changed at different time points, which may be related to differences in the gene levels caused by the different stages of bacterial growth (Huang et al., 2004). Rutin could reduce gene expression, but had no effect on the protein level at this stage. This may be due to a variety of factors, including low level of protein expression, sample processing, and the actual relationship between transcript and protein abundance (Pascal et al., 2008; Vogel and Marcotte, 2012; Schwanhaeusser et al., 2013; Cheng et al., 2016; Chick et al., 2016; Liu et al., 2016; Lau et al., 2018).

### Determination of Histidine Content

IGPD is one of the most important metalloenzymes in L-histidine biosynthesis (Carsiotis and Jones, 1974; Chiariotti et al., 1986; Gohda et al., 1998; Bisson et al., 2015). When IGPD was inhibited, its histidine content also decreased (Kulis-Horn et al., 2014; Zhou et al., 2018). Consequently, we used HPLC to determine the effect of rutin (0.8 mg/ml) on the histidine content of *S. xylosus* at 6, 12 and 24 h, respectively. Rutin significantly reduced the content of L-histidine at different time points (Figure 3C). Similarly, we also postulated that the content of L-histidine may also be affected by the metabolic pathways. Again, to further substantiate this hypothesis, we examined the effect of rutin (0.8 mg/ml) on the content of L-histidine in the *S. xylosus ΔhisB*. The results showed that there was no significant difference between the two groups (Figure 3D). This indicated that the effect of rutin on the L-histidine content of *S. xylosus* was only caused by its interference with the activity of IGPD and thereby inhibiting its biosynthesis.

As we have previously analyzed, rutin inhibited the activity of IGPD by binding to its active site, leading to a decrease in L-histidine biosynthesis, thereby interfering with the formation of biofilm by *S. xylosus*. In addition, L-histidine also can negative feedback regulation the expression of *hisB* gene. When bacteria are deprived of L-histidine, their *hisB* gene level will rise to cope with this change (Busch et al., 2001). However, our results suggest that rutin simultaneously inhibited the activity of IGPD and the expression of *hisB* gene. As a result of this, the bacteria could not synthesize their L-histidine by themselves, thereby leading to a significant reduction in their histidine content.

### Protective Effect of Rutin on Mice Infected With *S. xylosus*

**FIGURE 4:**
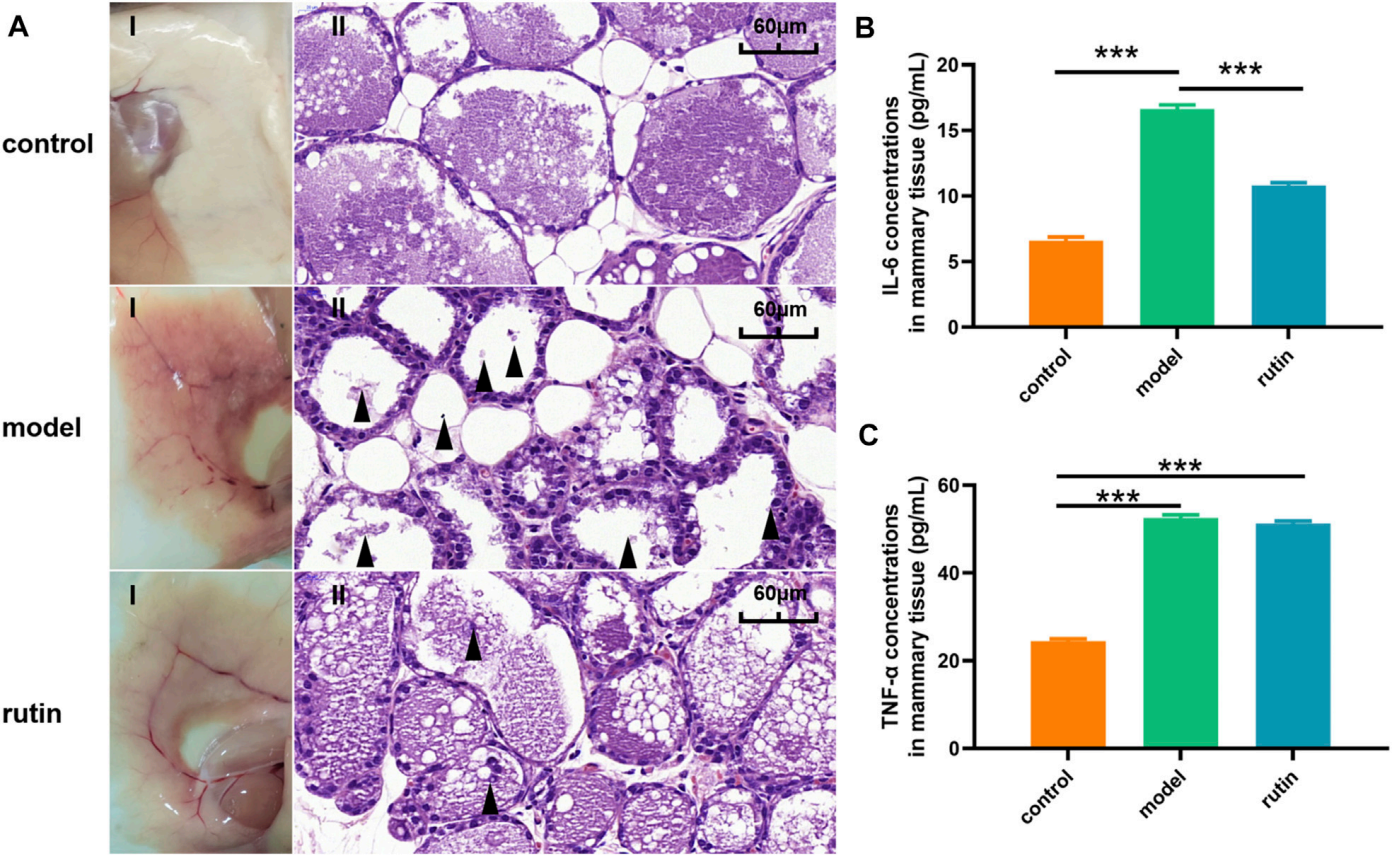
*In vivo* determination of the effect of rutin on the virulence of *S. xylosus* **. (A):** The gross pathological changes (I) and histopathological analysis (II) of the mammary gland tissues at 48 h post-infection (*n* = 5) (Magnification: ×600). The model group showed a large number of inflammatory factors and breast tissue destruction (arrowheads). The addition of rutin significantly improved the symptoms. **(B and C):** The levels of cytokines, including IL-6, and TNF-α, in the mammary gland tissues of infected mice were evaluated by ELISA (*n* = 5). The asterisk indicates statistical significance by *t* test with *p* < *0.05*.

In addition, we evaluated whether the presence of rutin attenuated the virulence of *S. xylosus* in a mouse mastitis model. After a careful investigation, it was observed that the mammary glands in the infected group showed morphological and pathological changes including inflammation and bleeding (Figure 4A). This was similar to what was observed in previous studies (Lai et al., 2017; Wang et al., 2018). Meanwhile, the intervention of rutin resulted in the attenuation of the effect of *S. xylosus* on the mammary tissues. Damaged tissues were significantly remedied and revitalized, the epithelial cell structures and morphology were completely restored, and the inflammation and bleeding reduced. There was no observable change in the control group. As we know, bacterial biofilm is closely related to its virulence Wroe et al. (2020), and 0.8 mg/ml rutin can interfere with the formation of biofilm, thus effectively reducing the damage of breast tissue caused by *S. xylosus* infection and protecting mice from mastitis. At the same time, the production of inflammation will cause immune response, release a variety of inflammatory factors including IL-6 and TNF - α, and cause damage to the body (Kanangat et al., 2001; Burmanczuk et al., 2018). Therefore, inhibiting the production of IL-6 and TNF - α will prevent inflammation at the same time. Furthermore, IL-6 is a pleiotropic cytokine, which is involved in the physiological activities of almost every organ in the biological system (Kimura and Kishimoto, 2010). Recent studies have shown that rutin can reduce the production of IL-6 in keratinocytes (di Giacomo et al., 2021). Our study showed that rutin significantly reduced the content of IL-6 in mouse mammary gland (Figure 4B). This may be related to the inhibition of Th2 mediated immune response (Zhao et al., 2015). Interestingly, rutin could not reduce the level of TNF-α, which may be the reason why rutin could not inhibit the growth of the bacteria (Figure 4C). At the same time, TNF - *α* can also induce neutrophils to chemotaxis, which may be one of the ways for animals to protect themselves (Okuda et al., 1997).

## Conclusion

*S. xylosus* isolated from some clinically infectious cases have gained interest in recent years, such as erythema nodosum Giordano et al. (2016), pyelonephritis Tselenis-Kotsowilis et al. (1982) and corneal infections (Makki et al., 2011). Even if the role of *S. xylosus* in these infections is uncertain, the biofilm formed by *S. xylosus* makes these infections pathogenically more chronic and difficult to cure because under such biofilm protection, the bacteria have greater chance of prolong survival in the host, thus rendering the intervention of therapeutic drugs insignificant. Thus, there is an urgent need to find an alternative and more effective strategy to interfere with the process of biofilm formation in pathogenic bacteria such as *S. xylosus* so as to abrogate their pathogenesis in living hosts. Here we discovered an inhibitor against IGPD, that can directly bind to IGPD, which makes it unable to dehydrate IGP to IAP. At the same time, it can down regulate the expression of *hisB* mRNA and inhibit the IGPD synthesis. Eventually, leading to the decrease of histidine content, which interferes with the initial adhesion of the biofilm of *S. xylosus,* thereby partially inhibiting the biofilm formation *in vitro* and weakening its virulence *in vivo*. From the foregoing therefore, the mechanism by which rutin interfered with the formation of biofilm in *S. xylosus* was well elucidated, and novel insights for controlling biofilm mediated persistent and chronic bacterial infections were provided.

## Data Availability

The original contributions presented in the study are included in the article/Supplementary Material, further inquiries can be directed to the corresponding authors.

## References

[B1] Al-Harbi N. O. Imam F. Al-Harbi M. M. Al-Shabanah O. A. Alotaibi M. R. As Sobeai H. M. (2019). Rutin Inhibits Carfilzomib-Induced Oxidative Stress and Inflammation via the NOS-Mediated NF-Κb Signaling Pathway. Inflammopharmacol 27 (4), 817–827. 10.1007/s10787-018-0550-5 30600471

[B2] Al-Shabib N. A. Husain F. M. Ahmad I. Khan M. S. Khan R. A. Khan J. M. (2017). Rutin Inhibits Mono and Multi-Species Biofilm Formation by Foodborne Drug Resistant *Escherichia coli* and *Staphylococcus aureus* . Food Control 79, 325–332. 10.1016/j.foodcont.2017.03.004

[B3] Alifano P. Fani R. Liò P. Lazcano A. Bazzicalupo M. Carlomagno M. S. (1996). Histidine Biosynthetic Pathway and Genes: Structure, Regulation, and Evolution. Microbiol. Rev. 60 (1), 44–69. 10.1128/mmbr.60.1.44-69.1996 8852895 PMC239417

[B4] Attaran B. Falsafi T. Ghorbanmehr N. (2017). Effect of Biofilm Formation by Clinical Isolates ofHelicobacter Pylorion the Efflux-Mediated Resistance to Commonly Used Antibiotics. Wjg 23 (7), 1163–1170. 10.3748/wjg.v23.i7.1163 28275296 PMC5323441

[B5] Bai J. Yang Y. Wang S. Gao L. Chen J. Ren Y. (2017). Syringa Oblata Lindl. Aqueous Extract Is a Potential Biofilm Inhibitor in S. Suis. Front. Pharmacol. 8. 10.3389/fphar.2017.00026 PMC5278344 28194111

[B6] Bisson C. Britton K. L. Sedelnikova S. E. Rodgers H. F. Eadsforth T. C. Viner R. C. (2015). Crystal Structures Reveal that the Reaction Mechanism of Imidazoleglycerol-Phosphate Dehydratase Is Controlled by Switching Mn(II) Coordination. Structure 23 (7), 1236–1245. 10.1016/j.str.2015.05.012 26095028 PMC4509728

[B7] Bochniarz M. Wawron W. Szczubiał M. Brodzki P. Piech T. Kusy R. (2014). CHARACTERISTICS OF STAPHYLOCOCCUS XYLOSUS ISOLATED FROM SUBCLINICAL MASTITIS IN COWS. Ann. Anim. Sci. 14 (4), 859–867. 10.2478/aoas-2014-0053

[B8] Bou Zeidan M. Zara G. Viti C. Decorosi F. Mannazzu I. Budroni M. (2014). L-histidine Inhibits Biofilm Formation and FLO11-Associated Phenotypes in *Saccharomyces cerevisiae* Flor Yeasts. Plos One 9 (11), e112141. 10.1371/journal.pone.0112141 25369456 PMC4219837

[B9] Bowers K. J. Chow D. E. Xu H. Dror R. O. Eastwood M. P. Gregersen B. A. (2006). "Scalable Algorithms for Molecular Dynamics Simulations on Commodity Clusters", in: SC '06: Proceedings of the 2006 ACM/IEEE Conference on Supercomputing, 43.

[B10] Breser M. L. Felipe V. Bohl L. P. Orellano M. S. Isaac P. Conesa A. (2018). Chitosan and Cloxacillin Combination Improve Antibiotic Efficacy against Different Lifestyle of Coagulase-Negative Staphylococcus Isolates from Chronic Bovine Mastitis. Sci. Rep. 8. 10.1038/s41598-018-23521-0 PMC5865155 29572457

[B11] Burmańczuk A. Hola P. Milczak A. Piech T. Kowalski C. Wojciechowska B. (2018). Quercetin Decrease Somatic Cells Count in Mastitis of Dairy Cows. Res. Vet. Sci. 117, 255–259. 10.1016/j.rvsc.2018.01.006 29331686

[B12] Busch S. Hoffmann B. Valerius O. Starke K. Düvel K. Braus G. H. (2001). Regulation of the Aspergillus nidulans hisB Gene by Histidine Starvation. Curr. Genet. 38 (6), 314–322. 10.1007/s002940000171 11270573

[B13] Carsiotis M. Jones R. F. (1974). Cross-pathway Regulation: Tryptophan-Mediated Control of Histidine and Arginine Biosynthetic Enzymes in Neurospora Crassa. J. Bacteriol. 119 (3), 889–892. 10.1128/jb.119.3.889-892.1974 4368541 PMC245695

[B14] Chen X.-R. Liu Y.-Y. Zhou Y.-H. Xing X.-X. Qu Q.-W. Chen X.-Y. (2019). Process Optimization of Syringa Oblata Lindl. By Response Surface Methodology and its Effect on Staphylococcus Xylosus Biofilm. RSC Adv. 9 (62), 36088–36096. 10.1039/c9ra06224f PMC9074935 35540619

[B15] Chen X.-R. Wang X.-T. Hao M.-Q. Zhou Y.-H. Cui W.-Q. Xing X.-X. (2017). Homology Modeling and Virtual Screening to Discover Potent Inhibitors Targeting the Imidazole Glycerophosphate Dehydratase Protein in Staphylococcus Xylosus. Front. Chem. 5. 10.3389/fchem.2017.00098 PMC5686052 29177138

[B16] Cheng Z. Teo G. Krueger S. Rock T. M. Koh H. W. Choi H. (2016). Differential Dynamics of the Mammalian mRNA and Protein Expression Response to Misfolding Stress. Mol. Syst. Biol. 12 (1), 855. 10.15252/msb.20156423 26792871 PMC4731011

[B17] Chiariotti L. Nappo A. G. Carlomagno M. S. Bruni C. B. (1986). Gene Structure in the Histidine Operon of *Escherichia coli* . Mol. Gen. Genet. 202 (1), 42–47. 10.1007/bf00330514 3007936

[B18] Chick J. M. Munger S. C. Simecek P. Huttlin E. L. Choi K. Gatti D. M. (2016). Defining the Consequences of Genetic Variation on a Proteome-wide Scale. Nature 534(7608)**,** 500, 505-+. 10.1038/nature18270 27309819 PMC5292866

[B19] Conrad J. C. Poling-Skutvik R. (2018). Confined Flow: Consequences and Implications for Bacteria and Biofilms Annu. Rev. Chem. Biomol. Eng. 9, 175–200. 10.1146/annurev-chembioeng-060817-084006 29561646

[B20] di Giacomo V. Recinella L. Chiavaroli A. Orlando G. Cataldi A. Rapino M. (2021). Metabolomic Profile and Antioxidant/Anti-Inflammatory Effects of Industrial Hemp Water Extract in Fibroblasts, Keratinocytes and Isolated Mouse Skin Specimens. Antioxidants 10 (1), 44. 10.3390/antiox10010044 PMC7823476 33401488

[B21] Fu Y. Sun Y.-N. Yi K.-H. Li M.-Q. Cao H.-F. Li J.-Z. (2018). Combination of Virtual Screening Protocol by In Silico toward the Discovery of Novel 4-Hydroxyphenylpyruvate Dioxygenase Inhibitors. Front. Chem. 6. 10.3389/fchem.2018.00014 PMC5807903 29468151

[B22] Giordano N. Corallo C. Miracco C. Papakostas P. Montella A. Figura N. (2016). Erythema Nodosum Associated with Staphylococcus Xylosus Septicemia. J. Microbiol. Immunol. Infect. 49 (1), 134–137. 10.1016/j.jmii.2012.10.003 23266237

[B23] Gohda K. Kimura Y. Mori I. Ohta D. Kikuchi T. (1998). Theoretical Evidence of the Existence of a Diazafulvene Intermediate in the Reaction Pathway of Imidazole glycerol Phosphate Dehydratase: Design of a Novel and Potent Heterocycle Structure for the Inhibitor on the Basis of the Electronic Structure-Activity Relationship Study. Biochim. Biophys. Acta (Bba) - Protein Struct. Mol. Enzymol. 1385 (1), 107–114. 10.1016/s0167-4838(98)00049-1 9630553

[B24] Grossmann M. Szkudlinski M. W. Dias J. A. Xia H. Wong R. Puett D. (1996). Site-directed Mutagenesis of Amino Acids 33-44 of the Common Alpha- Subunit Reveals Different Structural Requirements for Heterodimer Expression Among the Glycoprotein Hormones and Suggests that Cyclic Adenosine 3',5'-monophosphate Production and Growth Promotion Are Potentially Dissociable Functions of Human Thyrotropin. Mol. Endocrinol. 10 (6), 769–779. 10.1210/me.10.6.769 8776737

[B25] Hilton J. L. Kearney P. C. Ames B. N. (1965). Mode of Action of the Herbicide, 3-Amino-1,2,4-Triazole(amitrole): Inhibition of an Enzyme of Histidine Biosynthesis. Arch. Biochem. Biophys. 112 (3), 544–547. 10.1016/0003-9861(65)90093-7 5326242

[B26] Huang X. Pan W. Park S. Han X. Miller L. W. Hall J. (2004). Modeling the Relationship between LVAD Support Time and Gene Expression Changes in the Human Heart by Penalized Partial Least Squares. Bioinformatics 20 (6), 888–894. 10.1093/bioinformatics/btg499 14751963

[B27] Kanangat S. Bronze M. S. Meduri G. U. Postlethwaite A. Stentz F. Tolley E. (2001). Enhanced Extracellular Growth ofStaphylococcus Aureusin the Presence of Selected Linear Peptide Fragments of Human Interleukin (IL)-1β and IL‐1 Receptor Antagonist. J. Infect. Dis. 183 (1), 65–69. 10.1086/317645 11076706

[B28] Kimura A. Kishimoto T. (2010). IL-6: Regulator of Treg/Th17 Balance. Eur. J. Immunol. 40 (7), 1830–1835. 10.1002/eji.201040391 20583029

[B29] Korkmaz A. Kolankaya D. (2010). Protective Effect of Rutin on the Ischemia/Reperfusion Induced Damage in Rat Kidney. J. Surg. Res. 164 (2), 309–315. 10.1016/j.jss.2009.03.022 19592016

[B30] Kulis-Horn R. K. Persicke M. Kalinowski J. (2014). Histidine Biosynthesis, its Regulation and Biotechnological Application in Corynebacterium Glutamicum. Microb. Biotechnol. 7 (1), 5–25. 10.1111/1751-7915.12055 23617600 PMC3896937

[B31] Lai J.-l. Liu Y.-h. Peng Y.-c. Ge P. He C.-f. Liu C. (2017). Indirubin Treatment of Lipopolysaccharide-Induced Mastitis in a Mouse Model and Activity in Mouse Mammary Epithelial Cells. Mediators Inflamm. 2017, 1–13. 10.1155/2017/3082805 PMC5309412 28255203

[B32] Lau E. Cao Q. Lam M. P. Y. Wang J. Ng D. C. M. Bleakley B. J. (2018). Integrated Omics Dissection of Proteome Dynamics during Cardiac Remodeling. Nat. Commun. 9. 10.1038/s41467-017-02467-3 PMC5760723 29317621

[B33] Liu C. M. Hungate B. A. Tobian A. A. R. Serwadda D. Ravel J. Lester R. (2013). Male Circumcision Significantly Reduces Prevalence and Load of Genital Anaerobic Bacteria. Mbio 4 (2). 10.1128/mBio.00076-13 PMC3634604 23592260

[B34] Liu Y.-Y. Chen X.-R. Wang J.-P. Cui W.-Q. Xing X.-X. Chen X.-Y. (2019). Transcriptomic Analysis Reveals Flavonoid Biosynthesis of Syringa Oblata Lindl. In Response to Different Light Intensity. BMC Plant Biol. 19 (1). 10.1186/s12870-019-2100-8 PMC6849326 31711412

[B35] Liu Y. Beyer A. Aebersold R. (2016). On the Dependency of Cellular Protein Levels on mRNA Abundance. Cell 165 (3), 535–550. 10.1016/j.cell.2016.03.014 27104977

[B36] Lou Z. Tang Y. Song X. Wang H. (2015). Metabolomics-Based Screening of Biofilm-Inhibitory Compounds against *Pseudomonas aeruginosa* from Burdock Leaf. Molecules 20 (9), 16266–16277. 10.3390/molecules200916266 26370951 PMC6331861

[B37] Makki A. R. Sharma S. Duggirala A. Prashanth K. Garg P. Das T. (2011). Phenotypic and Genotypic Characterization of Coagulase Negative Staphylococci (CoNS) Other thanStaphylococcus epidermidisIsolated from Ocular Infections. Invest. Ophthalmol. Vis. Sci. 52 (12), 9018–9022. 10.1167/iovs.11-7777 22025577

[B38] Martin R. G. Goldberger R. F. (1967). Imidazolylacetolphosphate: L-Glutamate Aminotransferase. J. Biol. Chem. 242 (6), 1168–1174. 10.1016/s0021-9258(18)96159-4 5337155

[B39] Okuda A. Kubota M. Watanabe K. Sawada M. Koishi S. Kataoka A. (1997). Inhibition of Superoxide Production and Chemotaxis by Methotrexate in Neutrophils Primed by TNF-Alpha or LPS. Eur. J. Haematol. 59 (3), 142–147. 10.1111/j.1600-0609.1997.tb00967.x 9310121

[B40] Otero J. Baños R. González L. Torrents E. Juárez A. Puig-Vidal M. (2013). Quartz Tuning fork Studies on the Surface Properties of *Pseudomonas aeruginosa* during Early Stages of Biofilm Formation. Colloids Surf. B: Biointerfaces 102, 117–123. 10.1016/j.colsurfb.2012.08.013 23018019

[B41] Pascal L. E. True L. D. Campbell D. S. Deutsch E. W. Risk M. Coleman I. M. (2008). Correlation of mRNA and Protein Levels: Cell Type-specific Gene Expression of Cluster Designation Antigens in the Prostate. Bmc Genomics 9, 246. 10.1186/1471-2164-9-246 18501003 PMC2413246

[B42] Petersen J. Hawkes T. R. Lowe D. J. (1997). The Metal-Binding Site of Imidazole Glycerol Phosphate Dehydratase; EPR and ENDOR Studies of the Oxo-Vanadyl Enzyme. HEIDELBERG: JBIC.

[B43] Qu Q. Wang J. Cui W. Zhou Y. Xing X. Che R. (2019). *In Vitro* activity and *In Vivo* Efficacy of Isoliquiritigenin against Staphylococcus Xylosus ATCC 700404 by IGPD Target. Plos One 14 (12), e0226260. 10.1371/journal.pone.0226260 31860659 PMC6924684

[B44] Ragunathan A. Ravi L. (2015). Potential Antibacterial Drug Targets for Quercetin and Rutin: An In Silico Study Using AutoDock. Der Pharmacia Lettre 7 (11), 68–72.

[B45] Rawson S. Bisson C. Hurdiss D. L. Fazal A. McPhillie M. J. Sedelnikova S. E. (2018). Elucidating the Structural Basis for Differing Enzyme Inhibitor Potency by Cryo-EM. Proc. Natl. Acad. Sci. USA 115 (8), 1795–1800. 10.1073/pnas.1708839115 29434040 PMC5828572

[B46] Schleifer K. H. Kloos W. E. (1975). Isolation and Characterization of Staphylococci from Human Skin I. Amended Descriptions of Staphylococcus Epidermidis and Staphylococcus Saprophyticus and Descriptions of Three New Species: Staphylococcus Cohnii, Staphylococcus Haemolyticus, and Staphylococcus Xylosus. Int. J. Syst. Bacteriol. 25 (1), 50–61. 10.1099/00207713-25-1-50

[B47] Schwanhäusser B. Busse D. Li N. Dittmar G. Schuchhardt J. Wolf J. (2013). Corrigendum: Global Quantification of Mammalian Gene Expression Control. Nature 495 (7439), 126–127. 10.1038/nature11848 23407496

[B48] Szabados F. Strate K. Kaase M. Sakinc T. Anders A. Gatermann S. (2007). O177 Biofilm Formation May Be an Independent Virulence Factor in Wild-type Staphylococcus Saprophyticus Strain 7108 in Contrast to Wild-type Strain CCM 883. Int. J. Antimicrob. Agents 29, S36–S37. 10.1016/s0924-8579(07)70117-6

[B49] Tremblay Y. D. N. Hathroubi S. Jacques M. (2014). The Bacterian Biofilms - Their Importance in Animal Health and in Public Health -Yannick DN Tremblay. Can. J. Vet. Research-Revue Canadienne De Recherche Veterinaire 78 (2), 110–116. PMC3962273 24688172

[B50] Tselenis-Kotsowilis A. D. Koliomichalis M. P. Papavassiliou J. T. (1982). Acute Pyelonephritis Caused by Staphylococcus Xylosus. J. Clin. Microbiol. 16 (3), 593–594. 10.1128/jcm.16.3.593-594.1982 7130375 PMC272423

[B51] Velmourougane K. Prasanna R. (2017). Influence Ofl-Amino Acids on Aggregation and Biofilm Formation inAzotobacter chroococcumandTrichoderma Viride. J. Appl. Microbiol. 123 (4), 977–991. 10.1111/jam.13534 28731279

[B52] Vogel C. Marcotte E. M. (2012). Insights into the Regulation of Protein Abundance from Proteomic and Transcriptomic Analyses. Nat. Rev. Genet. 13 (4), 227–232. 10.1038/nrg3185 22411467 PMC3654667

[B53] Wang J. Li H. Pan J. Dong J. Zhou X. Niu X. (2018). Oligopeptide Targeting Sortase A as Potential Anti-infective Therapy for *Staphylococcus aureus* . Front. Microbiol. 9, 10. 10.3389/fmicb.2018.00245 29491861 PMC5817083

[B54] Wang S. Wang C. Gao L. Cai H. Zhou Y. Yang Y. (2017). Rutin Inhibits Streptococcus Suis Biofilm Formation by Affecting CPS Biosynthesis. Front. Pharmacol. 8, 12. 10.3389/fphar.2017.00379 28670278 PMC5472726

[B55] Woese C. R. (1987). Bacterial Evolution. Microbiol. Rev. 51 (2), 221–271. 10.1128/mmbr.51.2.221-271.1987 2439888 PMC373105

[B56] Wroe J. A. Johnson C. T. García A. J. (2020). Bacteriophage Delivering Hydrogels Reduce Biofilm Formation *In Vitro* and Infection *In Vivo* . J. Biomed. Mater. Res. 108 (1), 39–49. 10.1002/jbm.a.36790 PMC6880309 31443115

[B57] Xu C.-G. Yang Y.-B. Zhou Y.-H. Hao M.-Q. Ren Y.-Z. Wang X.-T. (2017). Comparative Proteomic Analysis Provides Insight into the Key Proteins as Possible Targets Involved in Aspirin Inhibiting Biofilm Formation of Staphylococcus Xylosus. Front. Pharmacol. 8. 10.3389/fphar.2017.00543 PMC5566577 28871227

[B58] Yang J. Guo S.-Y. Pan F.-Y. Geng H.-X. Gong Y. Lou D. (2007). Prokaryotic Expression and Polyclonal Antibody Preparation of a Novel Rab-like Protein mRabL5. Protein Expr. Purif. 53 (1), 1–8. 10.1016/j.pep.2006.10.025 17251037

[B59] Yang L. Liu Y. Wu H. Song Z. Høiby N. Molin S. (2012). Combating Biofilms. FEMS Immunol. Med. Microbiol. 65 (2), 146–157. 10.1111/j.1574-695X.2011.00858.x 22066868

[B60] Zhao Y. Zhou M. Gao Y. Liu H. Yang W. Yue J. (2015). Shifted T Helper Cell Polarization in a Murine *Staphylococcus aureus* Mastitis Model. Plos One 10 (7), e0134797. 10.1371/journal.pone.0134797 26230498 PMC4521801

[B61] Zhou Y.-h. Xu C.-g. Yang Y.-b. Xing X.-x. Liu X. Qu Q.-w. (2018). Histidine Metabolism and IGPD Play a Key Role in Cefquinome Inhibiting Biofilm Formation of Staphylococcus Xylosus. Front. Microbiol. 9 (665). 10.3389/fmicb.2018.00665 PMC5896262 29675012

