# Supplementary figures and images for: Rutin, A Natural Inhibitor of IGPD Protein, Partially Inhibits Biofilm Formation in Staphylococcus xylosus ATCC700404 in vitro and in vivo
Source: Front Pharmacol. 2021 Aug 11;12:728354. doi: 10.3389/fphar.2021.728354 (PMC8385535; doi:10.3389/fphar.2021.728354)

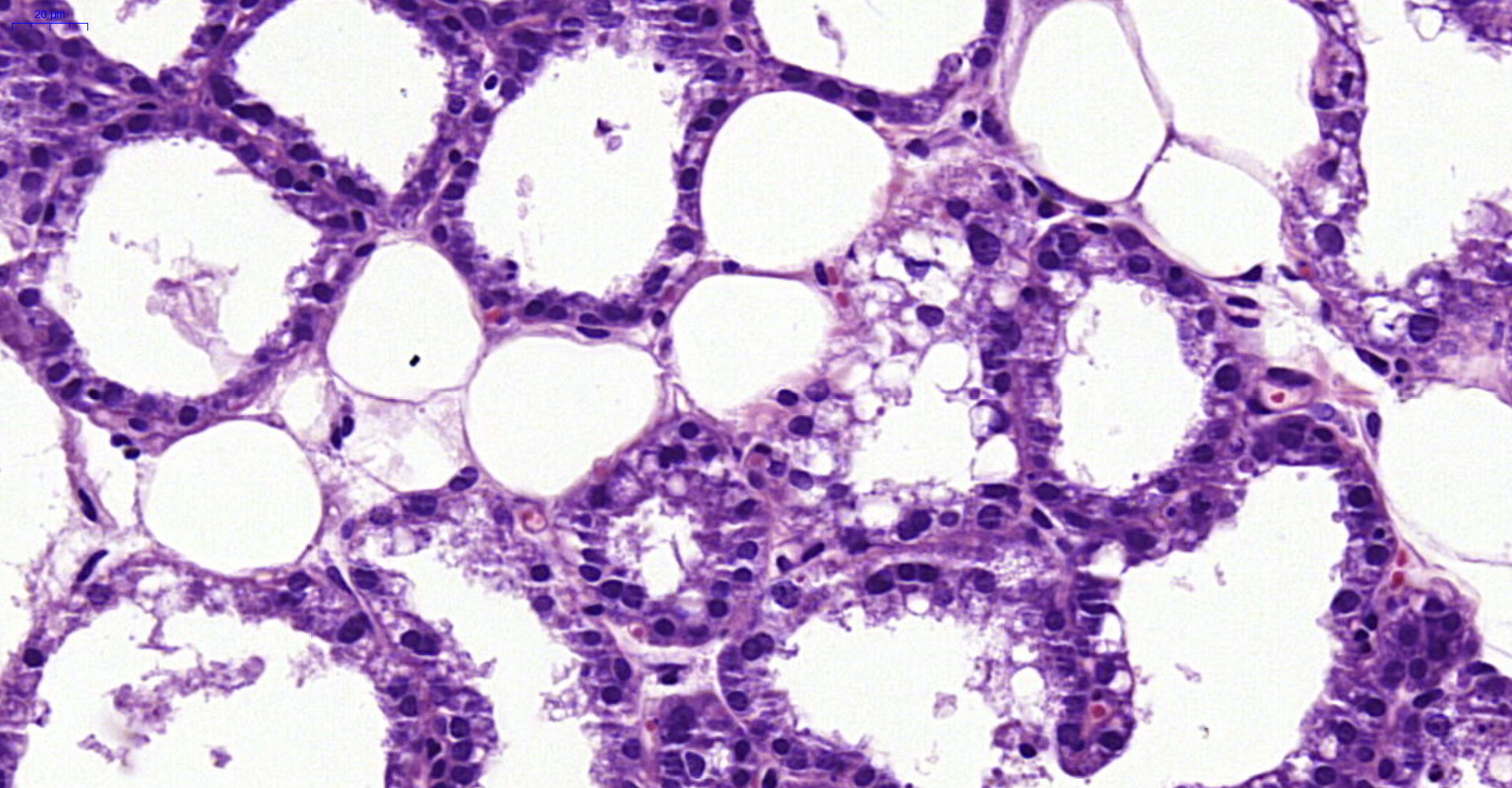

Supplement: Supplementary file 1 [file DataSheet3.zip › model_60.0x.jpg]

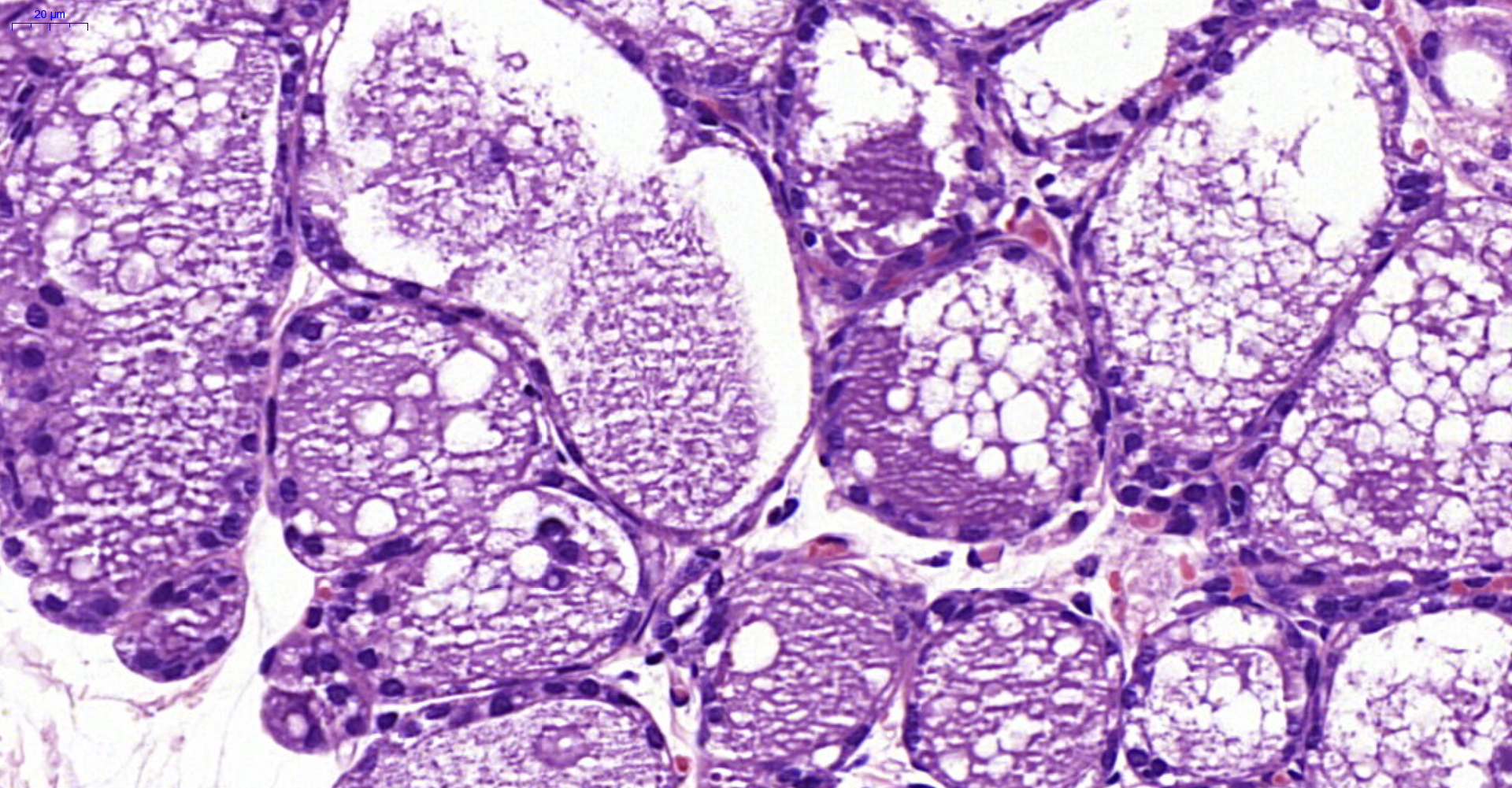

Supplement: Supplementary file 1 [file DataSheet3.zip › rutin_60.0x.jpg]

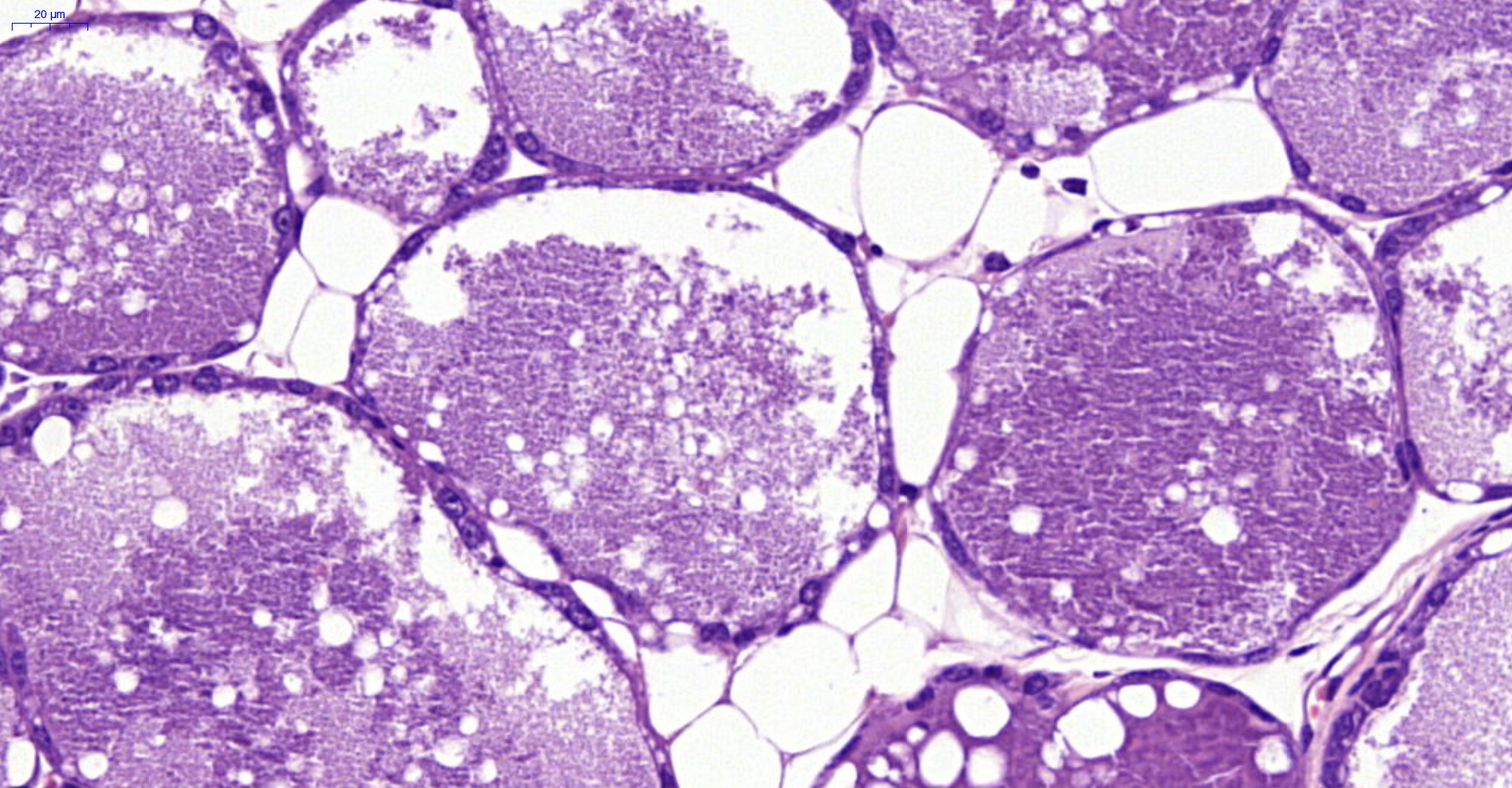

Supplement: Supplementary file 1 [file DataSheet3.zip › control_60.0x.jpg]

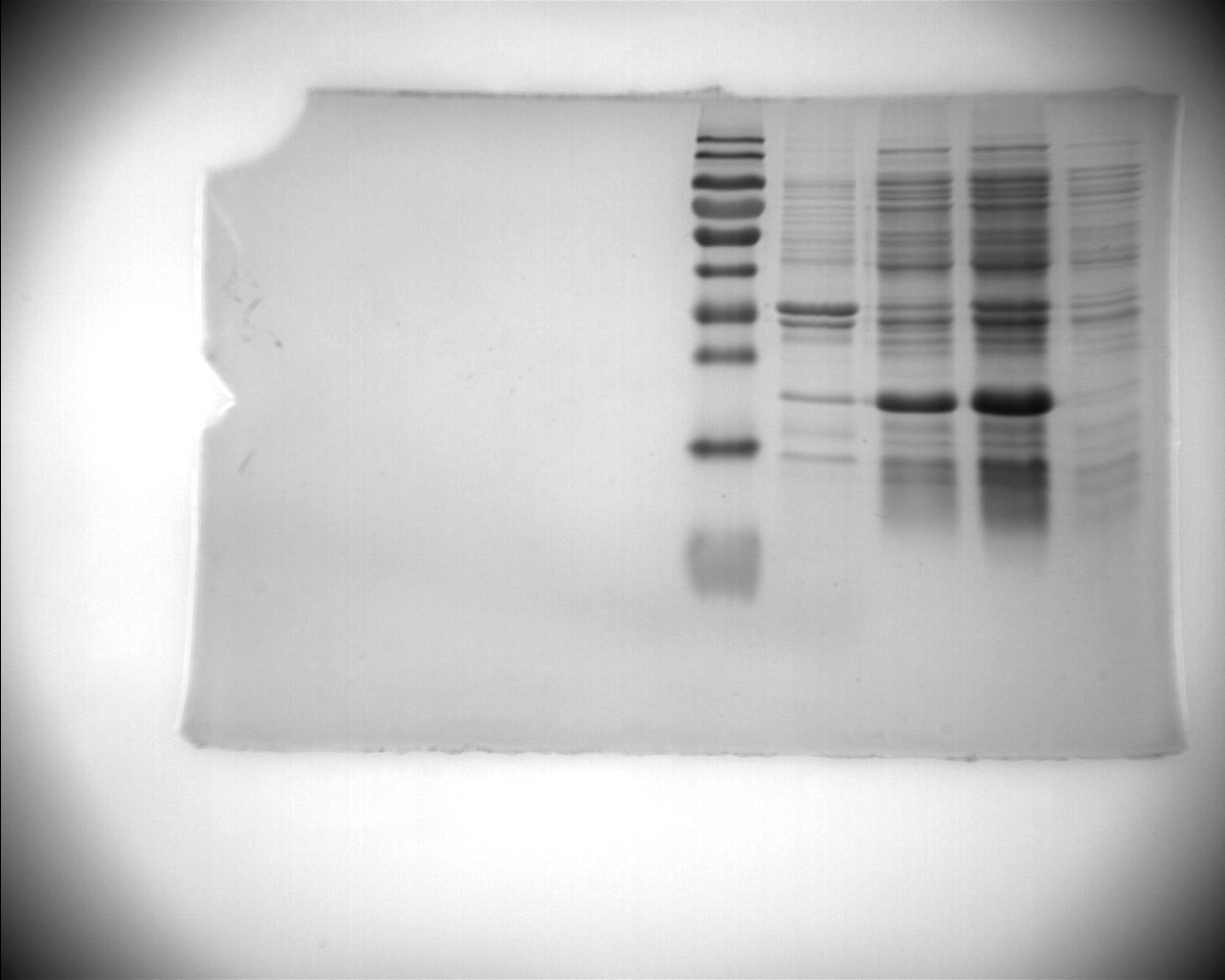

Supplement: Supplementary file 5 [file DataSheet2.zip › S4 Fig_A_raw_images.jpg]

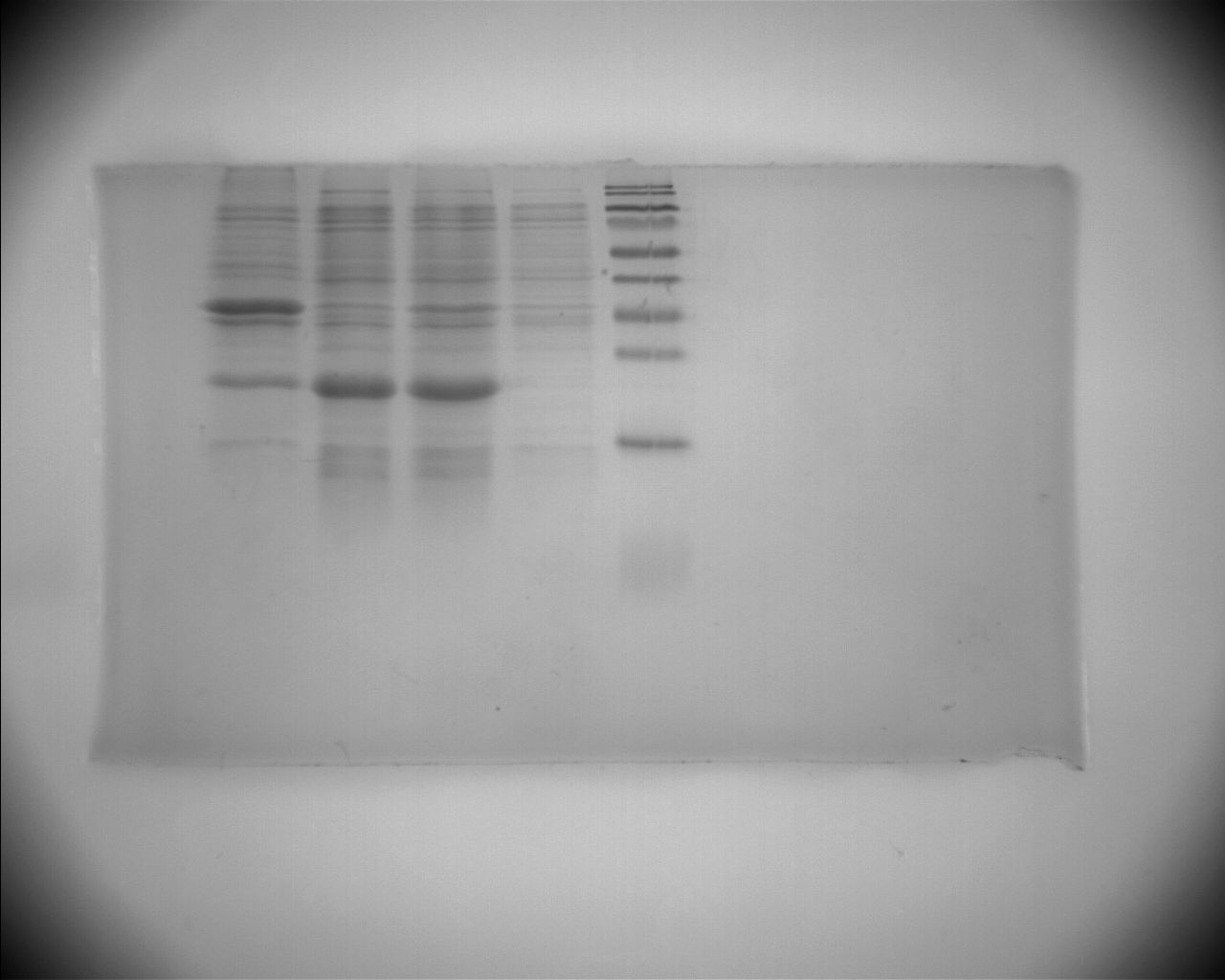

Supplement: Supplementary file 5 [file DataSheet2.zip › S4 Fig_B_raw_images.jpg]

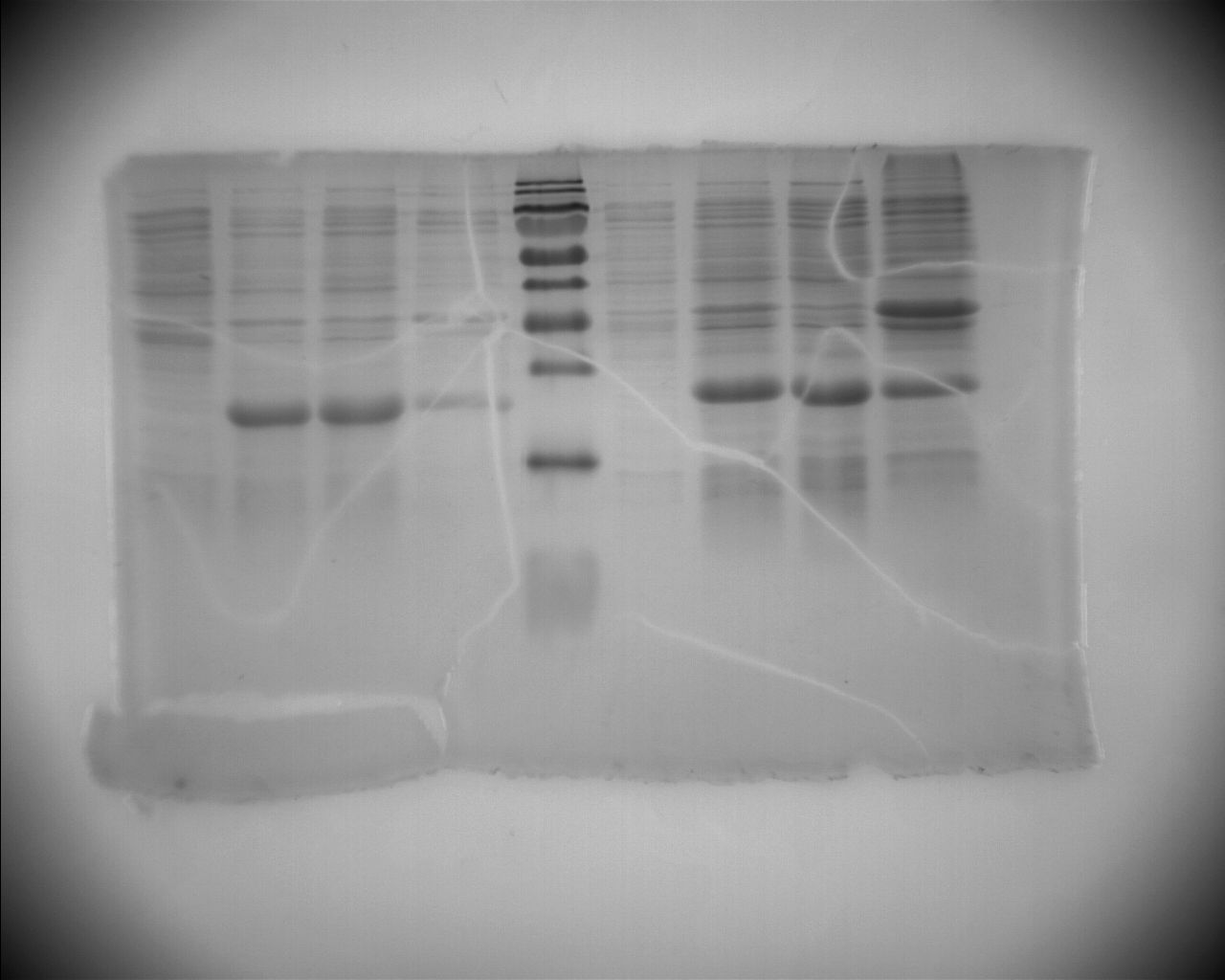

Supplement: Supplementary file 5 [file DataSheet2.zip › S4 Fig_D and C_raw_images.jpg]

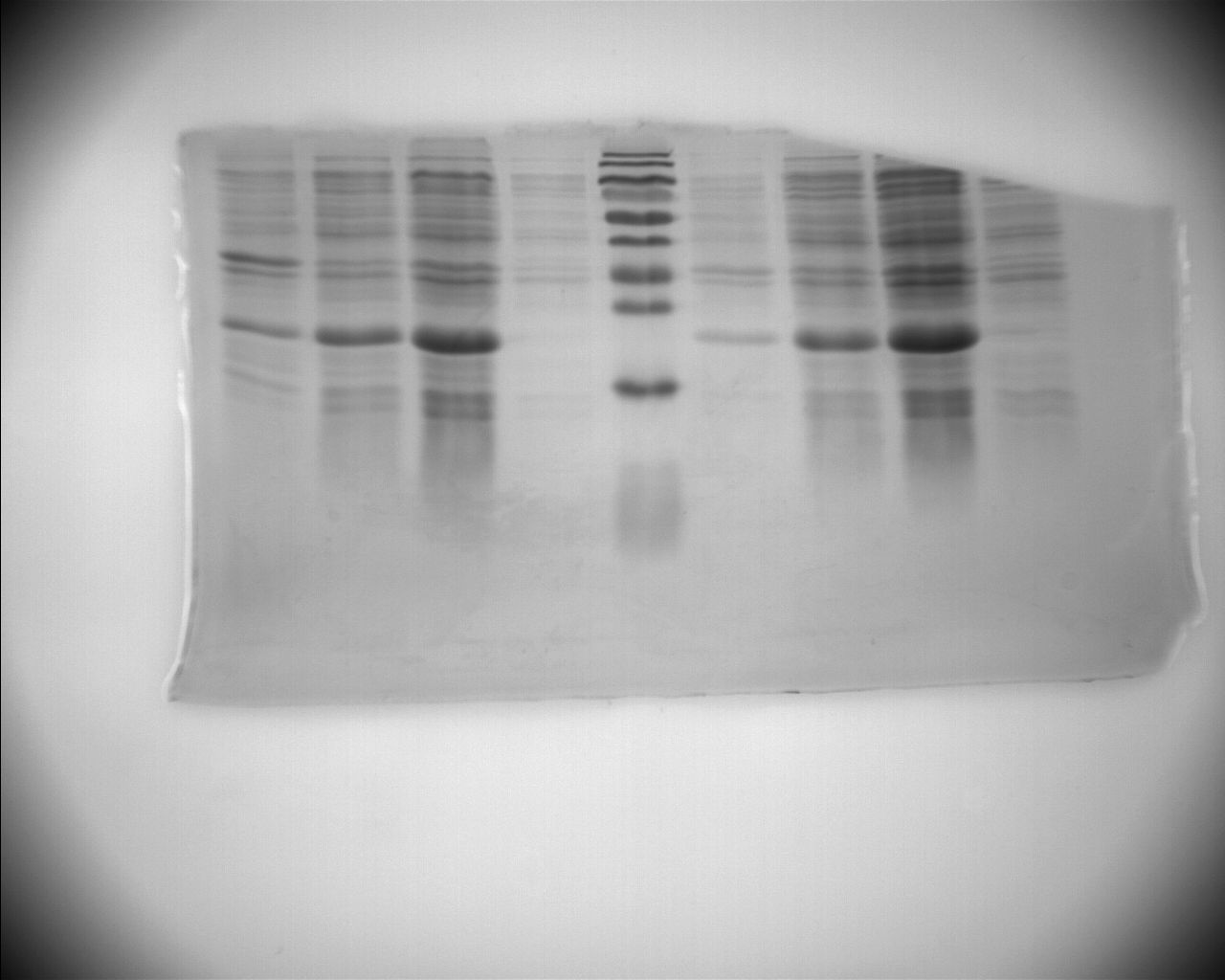

Supplement: Supplementary file 5 [file DataSheet2.zip › S4 Fig_E and F_raw_images.jpg]

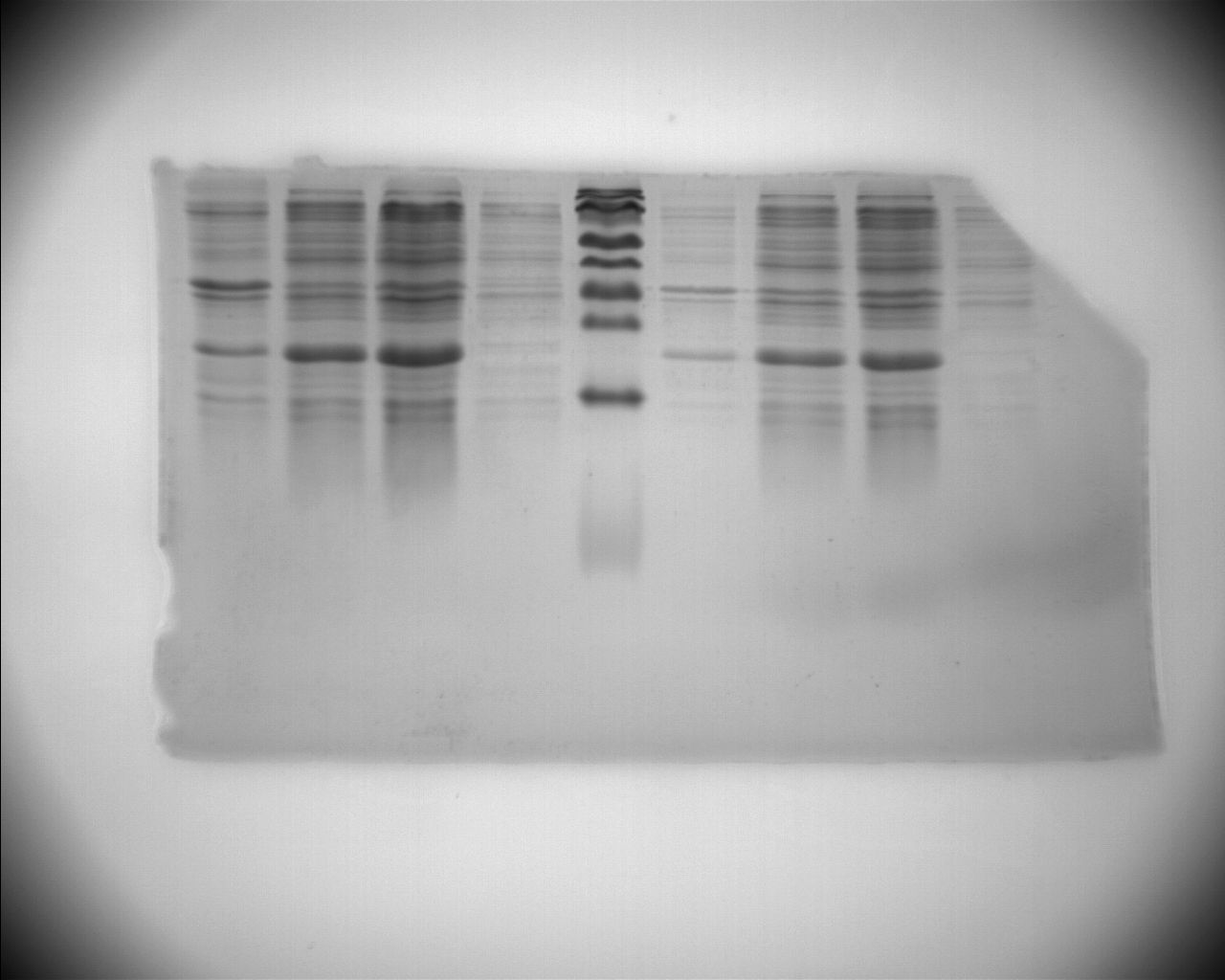

Supplement: Supplementary file 5 [file DataSheet2.zip › S4 Fig_G and H_raw_images.jpg]

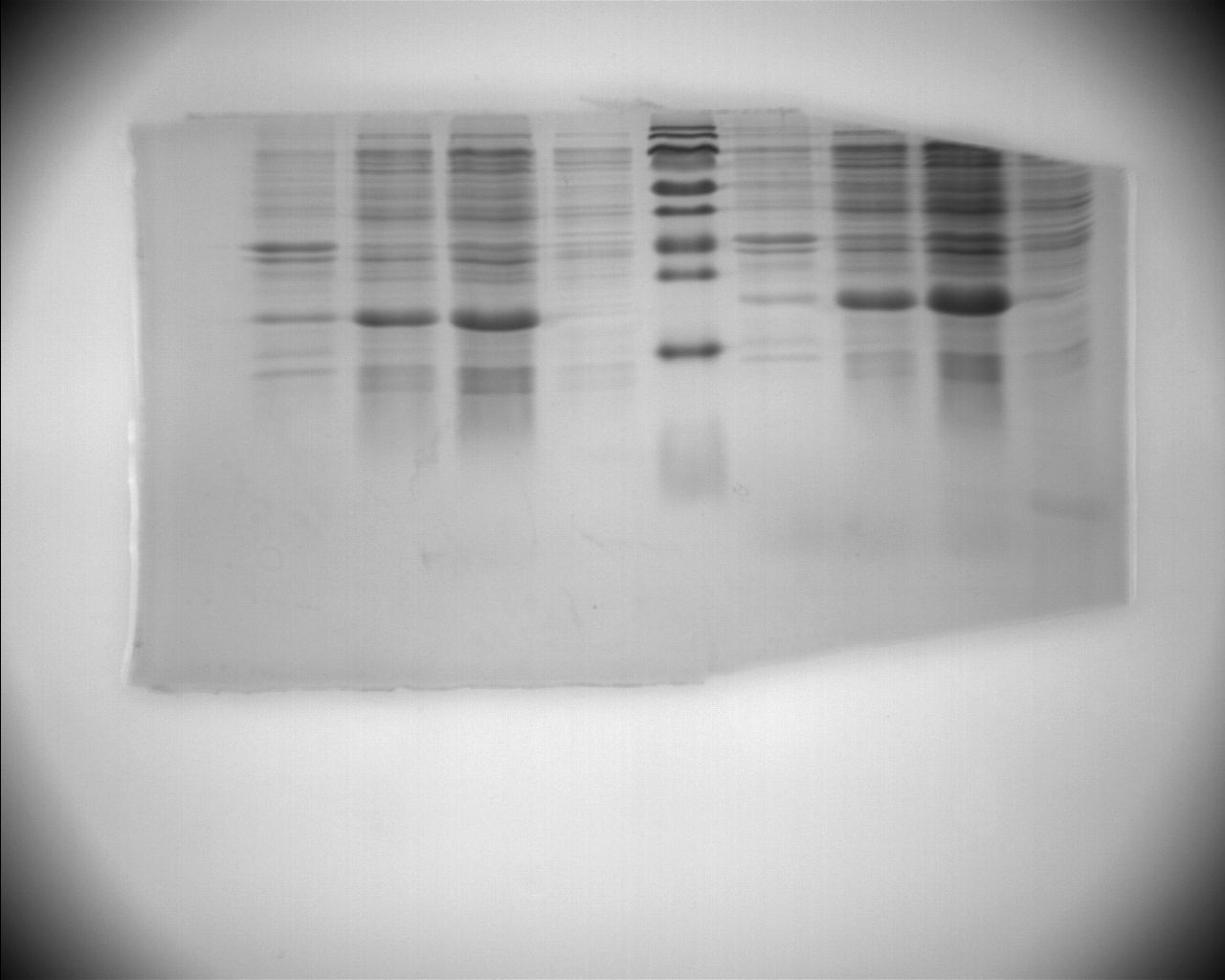

Supplement: Supplementary file 5 [file DataSheet2.zip › S4 Fig_I and J_raw_images.jpg]

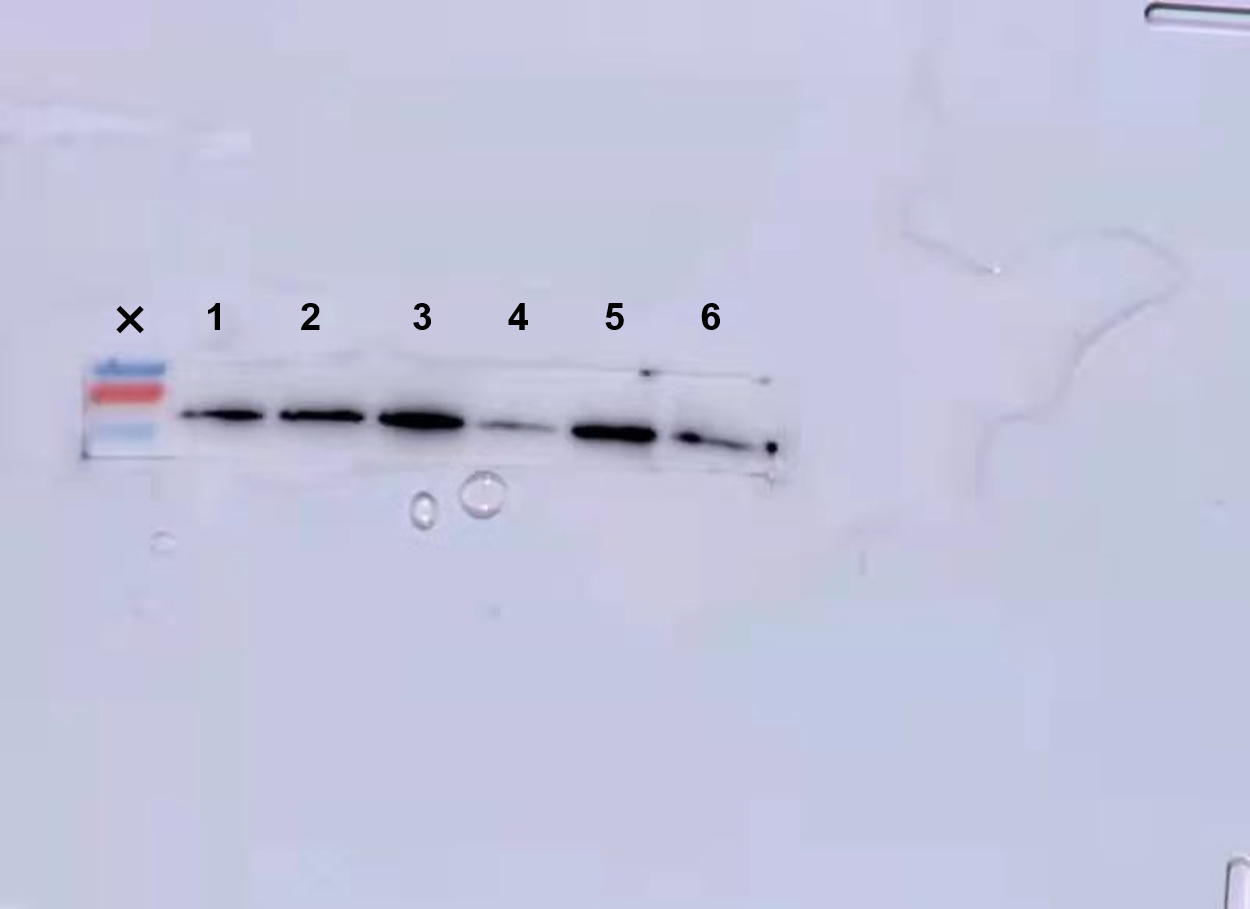

Supplement: Supplementary file 5 [file DataSheet2.zip › Fig3_B_raw_images.tif]
