# Supplementary material for: Rutin, A Natural Inhibitor of IGPD Protein, Partially Inhibits Biofilm Formation in Staphylococcus xylosus ATCC700404 in vitro and in vivo
Source: Front Pharmacol. 2021 Aug 11;12:728354. doi: 10.3389/fphar.2021.728354 (PMC8385535; doi:10.3389/fphar.2021.728354)
Supplement: Supplementary file 2 [file Table1.pdf]

Table 1. Bacterial strains and plasmids used in this study

Plasmids or strains	Description	Reference
**Strains**		
S. xylosus ATCC700404	Wild type strain	American Type Culture Collection
S. xylosus ΔhisB	Mutant strain	Lab stock [1]
E. coli DH5α	Used to amplify plasmids	Lab stock
E. coli BL21(DE3)	Used to expression of target proteins	Lab stock
**Plasmids**		
pET30a-IGPD	pET30a derivative for IGPD expression	Lab stock [1]
pET30a-Ala7	pET30a derivative for IGPD ΔArg7 expression	This study
pET30a-Ala62	pET30a derivative for IGPD ΔHis62 expression	This study
pET30a-Ala63	pET30a derivative for IGPD ΔHis63 expression	This study
pET30a-Ala66	pET30a derivative for IGPD ΔGlu66 expression	This study
pET30a-Ala97	pET30a derivative for IGPD ΔAsp97 expression	This study
pET30a-Ala110	pET30a derivative for IGPD ΔArg110 expression	This study
pET30a-Ala159	pET30a derivative for IGPD ΔHis159 expression	This study
pET30a-Ala162	pET30a derivative for IGPD ΔGlu162 expression	This study
pET30a-Ala166	pET30a derivative for IGPD ΔLys166 expression	This study

References

1. Zhou Y-h, Xu C-g, Yang Y-b, et al. Histidine Metabolism and IGPD Play a Key Role in Cefquinome Inhibiting Biofilm Formation of Staphylococcus xylosus [Original Research]. Frontiers in Microbiology. 2018 2018-April-05;9(665).
