# Supplementary material for: Rutin, A Natural Inhibitor of IGPD Protein, Partially Inhibits Biofilm Formation in Staphylococcus xylosus ATCC700404 in vitro and in vivo
Source: Front Pharmacol. 2021 Aug 11;12:728354. doi: 10.3389/fphar.2021.728354 (PMC8385535; doi:10.3389/fphar.2021.728354)
Supplement: Supplementary file 3 [file DataSheet4.zip › CG017-1 sequence alignment of añArg7 in pet30a IGPD .pdf]

```
                              ....|....| ....|....| ....|....| ....|....| ....|....|
                                      10         20         30         40         50
Ref of CG017-1                ---------- ---------- ---------- ---------- ----------
CG017-1-60_T7                 TCGTTACTCT AGAATAATTT TGTTTAACTT TAAGAAGGAG ATATACATAT

                              ....|....| ....|....| ....|....| ....|....| ....|....|
                                      60         70         80         90        100
Ref of CG017-1                -ATTTATCAA AAAACACGTA ACACTGCTGA AACACAACTA TCTATCTCAC
CG017-1-60_T7                 GATTTATCAA AAAACAGCAA ACACTGCTGA AACACAATTA TCTATCTCAC

                              ....|....| ....|....| ....|....| ....|....| ....|....|
                                     110        120        130        140        150
Ref of CG017-1                TTGCAGATGA CAATCGCCCA AGCAAAATCA ACACTGGCGT GGGTTTTCTA
CG017-1-60_T7                 TTGCAGATGA CAATCGCCCA AGCAAAATCA ACACTGGCGT GGGTTTTCTA

                              ....|....| ....|....| ....|....| ....|....| ....|....|
                                     160        170        180        190        200
Ref of CG017-1                GATCATATGT TGACCCTCTT CACCTTTCAT AGCAACTTAT CTATTACTAT
CG017-1-60_T7                 GATCATATGT TGACCCTCTT CACCTTTCAT AGCAACTTAT CTATTACTAT

                              ....|....| ....|....| ....|....| ....|....| ....|....|
                                     210        220        230        240        250
Ref of CG017-1                CGAAGCAAAT GGTGATACAG AAGTAGATGA TCACCACGTC ACAGAAGATA
CG017-1-60_T7                 CGAAGCAAAT GGTGATACAG AAGTAGACGA TCACCACGTC ACAGAAGATA

                              ....|....| ....|....| ....|....| ....|....| ....|....|
                                     260        270        280        290        300
Ref of CG017-1                TTGGTATTGT TTTAGGTCAA TTGTTGTTAG AAATGACTCG AGAAAGAAAA
CG017-1-60_T7                 TTGGTATTGT TTTAGGTCAA TTGTTGTTAG AAATGACTCG AGAAAGAAAA

                              ....|....| ....|....| ....|....| ....|....| ....|....|
                                     310        320        330        340        350
Ref of CG017-1                TCCTTTCAAC GTTATGGCGT AAGTTATATC CCTATGGATG AAACATTAGC
CG017-1-60_T7                 TCCTTTCAAC GTTATGGCGT AAGTTATATC CCTATGGATG AAACATTAGC

                              ....|....| ....|....| ....|....| ....|....| ....|....|
                                     360        370        380        390        400
Ref of CG017-1                ACGTACCGTC GTTGATATTA GTGGACGTCC TTTCCTTTCA TTTAATGCAC
CG017-1-60_T7                 ACGTACCGTC GTTGATATTA GTGGACGTCC TTTCCTTTCA TTTAATGCGC

                              ....|....| ....|....| ....|....| ....|....| ....|....|
                                     410        420        430        440        450
Ref of CG017-1                ATTTAAGCCG TGAAAAGGTA GGCACTTTTG ATACGGAATT AGTAGAAGAA
CG017-1-60_T7                 ATTTAAGTCG TGAAAAGGTA GGCACTTTTG ATACGGAATT AGTAGAAGAA

                              ....|....| ....|....| ....|....| ....|....| ....|....|
                                     460        470        480        490        500
Ref of CG017-1                TTCTTCCGTG CATTAGTCAT TAATGCACGC TTAACAACGC ATATTGATTT
CG017-1-60_T7                 TTCTTCCGTG CATTAGTCAT TAATGCCCGC TTAACAACGC ATATTGATTT

                              ....|....| ....|....| ....|....| ....|....| ....|....|
                                     510        520        530        540        550
Ref of CG017-1                AATACGTGGT GGTAATACCC ACCATGAAAT AGAAGGAATC TTCAAATCTT
CG017-1-60_T7                 AATACGTGGT GGTAATACTC ACCATGAAAT AGAAGGAATC TTCAAATCTT
```

```
                         ....|....| ....|....| ....|....| ....|....| ....|....|
                               560        570        580        590        600
Ref of CG017-1           TTGCGCGTGC ACTTAAAGAA TCTCTATCAA GCAATGACAT CGACGGCACG
CG017-1-60_T7            TTGCGCGTGC ACTTAAAGAA TCTCTATCAA GCAATGACAT CAACGGCACG

                         ....|....| ....|....| ....|....| ....|....| ....|....|
                               610        620        630        640        650
Ref of CG017-1           CCGTCATCTA AGGGTGTGAT AGAA------ ---------- ----------
CG017-1-60_T7            CCGTCATCTA AGGGTGTGAT AGAACTCGAG CACCACCACC ACCACCACTG

                         ....|....| ....|....| ....|....| ....|....| ....|....|
                               660        670        680        690        700
Ref of CG017-1           ---------- ---------- ---------- ---------- ----------
CG017-1-60_T7            AGATCCGGCT GCTAACAAAG CCCGAAAGGA AGCTGAGTTG GCTGCTGCCA

                         ....|....| ....|....| ....|....| ....|....| ....|....|
                               710        720        730        740        750
Ref of CG017-1           ---------- ---------- ---------- ---------- ----------
CG017-1-60_T7            CCGCTGAGCA ATAACTAGCA TAACCCCTTG GGGCCTCTAA ACGGGTCTTG

                         ....|....| ....|....| ....|....| ....|....| ....|....|
                               760        770        780        790        800
Ref of CG017-1           ---------- ---------- ---------- ---------- ----------
CG017-1-60_T7            AGGGGTTTTT TGCTGAAAGG AGGAACTATA TCCGGATTGG CGAATGGGAC

                         ....|....| ....|....| ....|....| ....|....| ....|....|
                               810        820        830        840        850
Ref of CG017-1           ---------- ---------- ---------- ---------- ----------
CG017-1-60_T7            GCGCCCTGTA GCGGCGCATT AAGCGCGGCG GGTGTGGTGG TTACGCGCAG

                         ....|....| ....|....| ....|....| ....|....| ....|....|
                               860        870        880        890        900
Ref of CG017-1           ---------- ---------- ---------- ---------- ----------
CG017-1-60_T7            CGTGACCGCT ACACTTGCCA GCGCCCTAGC GCCCGCTCCT TTCGCTTTCT

                         ....|....| ....|....| ....|....| ....|....| ....|....|
                               910        920        930        940        950
Ref of CG017-1           ---------- ---------- ---------- ---------- ----------
CG017-1-60_T7            TCCCTTCCTT TCTCGCCACG TTCGCCGGCT TTCCCCGTCA AGCTCTAAAT
```

Email: gene-syn@comatebio.com TEL: (+86)431-85871624 Web: www.comatebio.com
Add: 3333 ShengBei St., Building E1, Changchun City, Jilin Province, 130000
