# Supplementary material for: Rutin, A Natural Inhibitor of IGPD Protein, Partially Inhibits Biofilm Formation in Staphylococcus xylosus ATCC700404 in vitro and in vivo
Source: Front Pharmacol. 2021 Aug 11;12:728354. doi: 10.3389/fphar.2021.728354 (PMC8385535; doi:10.3389/fphar.2021.728354)
Supplement: Supplementary file 3 [file DataSheet4.zip › CG017-3 sequence alignment of añHis63 in pet30a IGPD .pdf]

```
                         ....|....| ....|....| ....|....| ....|....| ....|....|
                                10         20         30         40         50
Ref of CG017-3           ---------- ---------- ---------- ---------- ----------
CG017-3-114_T7           GGGGGGCGTA CTTCCTTCTA GAATAATTTT GTTTAACTTT AAGAAGGAGA

                         ....|....| ....|....| ....|....| ....|....| ....|....|
                                60         70         80         90        100
Ref of CG017-3           ---------- ATTTATCAAA AAACACGTAA CACTGCTGAA ACACAACTAT
CG017-3-114_T7           TATACATATG ATTTATCAAA AAACACGTAA CACTGCTGAA ACACAATTAT

                         ....|....| ....|....| ....|....| ....|....| ....|....|
                               110        120        130        140        150
Ref of CG017-3           CTATCTCACT TGCAGATGAC AATCGCCCAA GCAAAATCAA CACTGGCGTG
CG017-3-114_T7           CTATCTCACT TGCAGATGAC AATCGCCCAA GCAAAATCAA CACTGGCGTG

                         ....|....| ....|....| ....|....| ....|....| ....|....|
                               160        170        180        190        200
Ref of CG017-3           GGTTTTCTAG ATCATATGTT GACCCTCTTC ACCTTTCATA GCAACTTATC
CG017-3-114_T7           GGTTTTCTAG ATCATATGTT GACCCTCTTC ACCTTTCATA GCAACTTATC

                         ....|....| ....|....| ....|....| ....|....| ....|....|
                               210        220        230        240        250
Ref of CG017-3           TATTACTATC GAAGCAAATG GTGATACAGA AGTAGATGAT CACCACGTCA
CG017-3-114_T7           TATTACTATC GAAGCAAATG GTGATACAGA AGTAGATGAT CACGCAGTCA

                         ....|....| ....|....| ....|....| ....|....| ....|....|
                               260        270        280        290        300
Ref of CG017-3           CAGAAGATAT TGGTATTGTT TTAGGTCAAT TGTTGTTAGA AATGACTCGA
CG017-3-114_T7           CAGAAGATAT TGGTATTGTT TTAGGTCAAT TGTTGTTAGA AATGACTCGA

                         ....|....| ....|....| ....|....| ....|....| ....|....|
                               310        320        330        340        350
Ref of CG017-3           GAAAGAAAAT CCTTTCAACG TTATGGCGTA AGTTATATCC CTATGGATGA
CG017-3-114_T7           GAAAGAAAAT CCTTTCAACG TTATGGCGTA AGTTATATCC CTATGGATGA

                         ....|....| ....|....| ....|....| ....|....| ....|....|
                               360        370        380        390        400
Ref of CG017-3           AACATTAGCA CGTACCGTCG TTGATATTAG TGGACGTCCT TTCCTTTCAT
CG017-3-114_T7           AACATTAGCA CGTACCGTCG TTGATATTAG TGGACGTCCT TTCCTTTCAT

                         ....|....| ....|....| ....|....| ....|....| ....|....|
                               410        420        430        440        450
Ref of CG017-3           TTAATGCACA TTTAAGCCGT GAAAAGGTAG GCACTTTTGA TACGGAATTA
CG017-3-114_T7           TTAATGCGCA TTTAAGTCGT GAAAAGGTAG GCACTTTTGA TACGGAATTA

                         ....|....| ....|....| ....|....| ....|....| ....|....|
                               460        470        480        490        500
Ref of CG017-3           GTAGAAGAAT TCTTCCGTGC ATTAGTCATT AATGCACGCT TAACAACGCA
CG017-3-114_T7           GTAGAAGAAT TCTTCCGTGC ATTAGTCATT AATGCCCGCT TAACAACGCA

                         ....|....| ....|....| ....|....| ....|....| ....|....|
                               510        520        530        540        550
Ref of CG017-3           TATTGATTTA ATACGTGGTG GTAATACCCA CCATGAAATA GAAGGAATCT
CG017-3-114_T7           TATTGATTTA ATACGTGGTG GTAATACTCA CCATGAAATA GAAGGAATCT
```

```
                    ....|....| ....|....| ....|....| ....|....| ....|....|
                          560        570        580        590        600
Ref of CG017-3      TCAAATCTTT TGCGCGTGCA CTTAAAGAAT CTCTATCAAG CAATGACATC
CG017-3-114_T7      TCAAATCTTT TGCGCGTGCA CTTAAAGAAT CTCTATCAAG CAATGACATC

                    ....|....| ....|....| ....|....| ....|....| ....|....|
                          610        620        630        640        650
Ref of CG017-3      GACGGCACGC CGTCATCTAA GGGTGTGATA GAA------- ----------
CG017-3-114_T7      AACGGCACGC CGTCATCTAA GGGTGTGATA GAACTCGAGC ACCACCACCA

                    ....|....| ....|....| ....|....| ....|....| ....|....|
                          660        670        680        690        700
Ref of CG017-3      ---------- ---------- ---------- ---------- ----------
CG017-3-114_T7      CCACCACTGA GATCCGGCTG CTAACAAAGC CCGAAAGGAA GCTGAGTTGG

                    ....|....| ....|....| ....|....| ....|....| ....|....|
                          710        720        730        740        750
Ref of CG017-3      ---------- ---------- ---------- ---------- ----------
CG017-3-114_T7      CTGCTGCCAC CGCTGAGCAA TAACTAGCAT AACCCCTTGG GGCCTCTAAA

                    ....|....| ....|....| ....|....| ....|....| ....|....|
                          760        770        780        790        800
Ref of CG017-3      ---------- ---------- ---------- ---------- ----------
CG017-3-114_T7      CGGGTCTTGA GGGGTTTTTT GCTGAAAGGA GGAACTATAT CCGGATTGGC

                    ....|....| ....|....| ....|....| ....|....| ....|....|
                          810        820        830        840        850
Ref of CG017-3      ---------- ---------- ---------- ---------- ----------
CG017-3-114_T7      GAATGGGACG CGCCCTGTAG CGGCGCATTA AGCGCGGCGG GTGTGGTGGT

                    ....|....| ....|....| ....|....| ....|....| ....|....|
                          860        870        880        890        900
Ref of CG017-3      ---------- ---------- ---------- ---------- ----------
CG017-3-114_T7      TACGCGCAGC GTGACCGCTA CACTTGCCAG CGCCCTAGCG CCCGCTCCTT

                    ....|....| ....|....| ....|....| ....|....| ....|....|
                          910        920        930        940        950
Ref of CG017-3      ---------- ---------- ---------- ---------- ----------
CG017-3-114_T7      TCGCTTTCTT CCCTTCCTTT CTCGCCACGT TCGCCGGCTT TCCCCGTCAA
```
