# Supplementary material for: Rutin, A Natural Inhibitor of IGPD Protein, Partially Inhibits Biofilm Formation in Staphylococcus xylosus ATCC700404 in vitro and in vivo
Source: Front Pharmacol. 2021 Aug 11;12:728354. doi: 10.3389/fphar.2021.728354 (PMC8385535; doi:10.3389/fphar.2021.728354)
Supplement: Supplementary file 3 [file DataSheet4.zip › CG017-4 sequence alignment of añGlu66 in pet30a IGPD .pdf]

```
                           ....|....| ....|....| ....|....| ....|....| ....|....|
                                   10         20         30         40         50
Ref of CG017-4             ---------- ---------- ---------- ---------- ----------
CG017-4-121_T7             GTGGACGGTC TTCCATCTAG AATAATTTTG TTTAACTTTA AGAAGGAGAT

                           ....|....| ....|....| ....|....| ....|....| ....|....|
                                   60         70         80         90        100
Ref of CG017-4             ---------A TTTATCAAAA AACACGTAAC ACTGCTGAAA CACAACTATC
CG017-4-121_T7             ATACATATGA TTTATCAAAA AACACGTAAC ACTGCTGAAA CACAATTATC

                           ....|....| ....|....| ....|....| ....|....| ....|....|
                                  110        120        130        140        150
Ref of CG017-4             TATCTCACTT GCAGATGACA ATCGCCCAAG CAAAATCAAC ACTGGCGTGG
CG017-4-121_T7             TATCTCACTT GCAGATGACA ATCGCCCAAG CAAAATCAAC ACTGGCGTGG

                           ....|....| ....|....| ....|....| ....|....| ....|....|
                                  160        170        180        190        200
Ref of CG017-4             GTTTTCTAGA TCATATGTTG ACCCTCTTCA CCTTTCATAG CAACTTATCT
CG017-4-121_T7             GTTTTCTAGA TCATATGTTG ACCCTCTTCA CCTTTCATAG CAACTTATCT

                           ....|....| ....|....| ....|....| ....|....| ....|....|
                                  210        220        230        240        250
Ref of CG017-4             ATTACTATCG AAGCAAATGG TGATACAGAA GTAGATGATC ACCACGTCAC
CG017-4-121_T7             ATTACTATCG AAGCAAATGG TGATACAGAA GTAGATGATC ACCACGTCAC

                           ....|....| ....|....| ....|....| ....|....| ....|....|
                                  260        270        280        290        300
Ref of CG017-4             AGAAGATATT GGTATTGTTT TAGGTCAATT GTTGTTAGAA ATGACTCGAG
CG017-4-121_T7             AGCAGATATT GGTATTGTTT TAGGTCAATT GTTGTTAGAA ATGACTCGAG

                           ....|....| ....|....| ....|....| ....|....| ....|....|
                                  310        320        330        340        350
Ref of CG017-4             AAAGAAAATC CTTTCAACGT TATGGCGTAA GTTATATCCC TATGGATGAA
CG017-4-121_T7             AAAGAAAATC CTTTCAACGT TATGGCGTAA GTTATATCCC TATGGATGAA

                           ....|....| ....|....| ....|....| ....|....| ....|....|
                                  360        370        380        390        400
Ref of CG017-4             ACATTAGCAC GTACCGTCGT TGATATTAGT GGACGTCCTT TCCTTTCATT
CG017-4-121_T7             ACATTAGCAC GTACCGTCGT TGATATTAGT GGACGTCCTT TCCTTTCATT

                           ....|....| ....|....| ....|....| ....|....| ....|....|
                                  410        420        430        440        450
Ref of CG017-4             TAATGCACAT TTAAGCCGTG AAAAGGTAGG CACTTTTGAT ACGGAATTAG
CG017-4-121_T7             TAATGCGCAT TTAAGTCGTG AAAAGGTAGG CACTTTTGAT ACGGAATTAG

                           ....|....| ....|....| ....|....| ....|....| ....|....|
                                  460        470        480        490        500
Ref of CG017-4             TAGAAGAATT CTTCCGTGCA TTAGTCATTA ATGCACGCTT AACAACGCAT
CG017-4-121_T7             TAGAAGAATT CTTCCGTGCA TTAGTCATTA ATGCCCGCTT AACAACGCAT

                           ....|....| ....|....| ....|....| ....|....| ....|....|
                                  510        520        530        540        550
Ref of CG017-4             ATTGATTTAA TACGTGGTGG TAATACCCAC CATGAAATAG AAGGAATCTT
CG017-4-121_T7             ATTGATTTAA TACGTGGTGG TAATACTCAC CATGAAATAG AAGGAATCTT
```

Email: gene-syn@comatebio.com TEL: (+86)431-85871624 Web: www.comatebio.com
Add: 3333 ShengBei St., Building E1, Changchun City, Jilin Province, 130000

```
                                 ....|....| ....|....| ....|....| ....|....| ....|....|
                                        560        570        580        590        600
Ref of CG017-4                   CAAATCTTTT GCGCGTGCAC TTAAAGAATC TCTATCAAGC AATGACATCG
CG017-4-121_T7                   CAAATCTTTT GCGCGTGCAC TTAAAGAATC TCTATCAAGC AATGACATCA

                                 ....|....| ....|....| ....|....| ....|....| ....|....|
                                        610        620        630        640        650
Ref of CG017-4                   ACGGCACGCC GTCATCTAAG GGTGTGATAG AA-------- ----------
CG017-4-121_T7                   ACGGCACGCC GTCATCTAAG GGTGTGATAG AACTCGAGCA CCACCACCAC

                                 ....|....| ....|....| ....|....| ....|....| ....|....|
                                        660        670        680        690        700
Ref of CG017-4                   ---------- ---------- ---------- ---------- ----------
CG017-4-121_T7                   CACCACTGAG ATCCGGCTGC TAACAAAGCC CGAAAGGAAG CTGAGTTGGC

                                 ....|....| ....|....| ....|....| ....|....| ....|....|
                                        710        720        730        740        750
Ref of CG017-4                   ---------- ---------- ---------- ---------- ----------
CG017-4-121_T7                   TGCTGCCACC GCTGAGCAAT AACTAGCATA ACCCCTTGGG GCCTCTAAAC

                                 ....|....| ....|....| ....|....| ....|....| ....|....|
                                        760        770        780        790        800
Ref of CG017-4                   ---------- ---------- ---------- ---------- ----------
CG017-4-121_T7                   GGGTCTTGAG GGGTTTTTTG CTGAAAGGAG GAACTATATC CCGGATTGGC

                                 ....|....| ....|....| ....|....| ....|....| ....|....|
                                        810        820        830        840        850
Ref of CG017-4                   ---------- ---------- ---------- ---------- ----------
CG017-4-121_T7                   GAATGGGACG CGCCCTGTAG CGGCGCATTA AGCGCGGCGG GTGTGGTGGT

                                 ....|....| ....|....| ....|....| ....|....| ....|....|
                                        860        870        880        890        900
Ref of CG017-4                   ---------- ---------- ---------- ---------- ----------
CG017-4-121_T7                   TACGCGCAGC GTGACCGCTA CACTTGCCAG CGCCCTAGCG CCCGCTCCTT

                                 ....|....| ....|....| ....|....| ....|....| ....|....|
                                        910        920        930        940        950
Ref of CG017-4                   ---------- ---------- ---------- ---------- ----------
CG017-4-121_T7                   TCGCTTTCTT CCCTTCCTTT CTCGCCACGT TCGCCGGCTT TCCCCGTCAA
```

Email: gene-syn@comatebio.com TEL: (+86)431-85871624 Web: www.comatebio.com
Add: 3333 ShengBei St., Building E1, Changchun City, Jilin Province, 130000
