# Supplementary material for: Rutin, A Natural Inhibitor of IGPD Protein, Partially Inhibits Biofilm Formation in Staphylococcus xylosus ATCC700404 in vitro and in vivo
Source: Front Pharmacol. 2021 Aug 11;12:728354. doi: 10.3389/fphar.2021.728354 (PMC8385535; doi:10.3389/fphar.2021.728354)
Supplement: Supplementary file 3 [file DataSheet4.zip › CG017-5 sequence alignment of añAsp97 in pet30a IGPD .pdf]

```
                          ....|....| ....|....| ....|....| ....|....| ....|....|
                                  10         20         30         40         50
Ref of CG017-5            ---------- ---------- ---------- ---------- ----------
CG017-5-33_T7             GAGGTAATTC CTCTAGAATA ATTTTGTTTA ACTTTAAGAA GGAGATATAC

                          ....|....| ....|....| ....|....| ....|....| ....|....|
                                  60         70         80         90        100
Ref of CG017-5            -----ATTTA TCAAAAAACA CGTAACACTG CTGAAACACA ACTATCTATC
CG017-5-33_T7             ATATGATTTA TCAAAAAACA CGTAACACTG CTGAAACACA ATTATCTATC

                          ....|....| ....|....| ....|....| ....|....| ....|....|
                                 110        120        130        140        150
Ref of CG017-5            TCACTTGCAG ATGACAATCG CCCAAGCAAA ATCAACACTG GCGTGGGTTT
CG017-5-33_T7             TCACTTGCAG ATGACAATCG CCCAAGCAAA ATCAACACTG GCGTGGGTTT

                          ....|....| ....|....| ....|....| ....|....| ....|....|
                                 160        170        180        190        200
Ref of CG017-5            TCTAGATCAT ATGTTGACCC TCTTCACCTT TCATAGCAAC TTATCTATTA
CG017-5-33_T7             TCTAGATCAT ATGTTGACCC TCTTCACCTT TCATAGCAAC TTATCTATTA

                          ....|....| ....|....| ....|....| ....|....| ....|....|
                                 210        220        230        240        250
Ref of CG017-5            CTATCGAAGC AAATGGTGAT ACAGAAGTAG ATGATCACCA CGTCACAGAA
CG017-5-33_T7             CTATCGAAGC AAATGGTGAT ACAGAAGTAG ACGATCACCA CGTCACAGAA

                          ....|....| ....|....| ....|....| ....|....| ....|....|
                                 260        270        280        290        300
Ref of CG017-5            GATATTGGTA TTGTTTTAGG TCAATTGTTG TTAGAAATGA CTCGAGAAAG
CG017-5-33_T7             GATATTGGTA TTGTTTTAGG TCAATTGTTG TTAGAAATGA CTCGAGAAAG

                          ....|....| ....|....| ....|....| ....|....| ....|....|
                                 310        320        330        340        350
Ref of CG017-5            AAAATCCTTT CAACGTTATG GCGTAAGTTA TATCCCTATG GATGAAACAT
CG017-5-33_T7             AAAATCCTTT CAACGTTATG GCGTAAGTTA TATCCCTATG GCAGAAACAT

                          ....|....| ....|....| ....|....| ....|....| ....|....|
                                 360        370        380        390        400
Ref of CG017-5            TAGCACGTAC CGTCGTTGAT ATTAGTGGAC GTCCTTTCCT TTCATTTAAT
CG017-5-33_T7             TAGCACGTAC CGTCGTTGAT ATTAGTGGAC GTCCTTTCCT TTCATTTAAT

                          ....|....| ....|....| ....|....| ....|....| ....|....|
                                 410        420        430        440        450
Ref of CG017-5            GCACATTTAA GCCGTGAAAA GGTAGGCACT TTTGATACGG AATTAGTAGA
CG017-5-33_T7             GCGCATTTAA GTCGTGAAAA GGTAGGCACT TTTGATACGG AATTAGTAGA

                          ....|....| ....|....| ....|....| ....|....| ....|....|
                                 460        470        480        490        500
Ref of CG017-5            AGAATTCTTC CGTGCATTAG TCATTAATGC ACGCTTAACA ACGCATATTG
CG017-5-33_T7             AGAATTCTTC CGTGCATTAG TCATTAATGC CCGCTTAACA ACGCATATTG

                          ....|....| ....|....| ....|....| ....|....| ....|....|
                                 510        520        530        540        550
Ref of CG017-5            ATTTAATACG TGGTGGTAAT ACCCACCATG AAATAGAAGG AATCTTCAAA
CG017-5-33_T7             ATTTAATACG TGGTGGTAAT ACTCACCATG AAATAGAAGG AATCTTCAAA
```

```
                           ....|....| ....|....| ....|....| ....|....| ....|....|
                                  560        570        580        590        600
Ref of CG017-5             TCTTTTGCGC GTGCACTTAA AGAATCTCTA TCAAGCAATG ACATCGACGG
CG017-5-33_T7              TCTTTTGCGC GTGCACTTAA AGAATCTCTA TCAAGCAATG ACATCAACGG

                           ....|....| ....|....| ....|....| ....|....| ....|....|
                                  610        620        630        640        650
Ref of CG017-5             CACGCCGTCA TCTAAGGGTG TGATAGAA-- ---------- ----------
CG017-5-33_T7              CACGCCGTCA TCTAAGGGTG TGATAGAACT CGAGCACCAC CACCACCACC

                           ....|....| ....|....| ....|....| ....|....| ....|....|
                                  660        670        680        690        700
Ref of CG017-5             ---------- ---------- ---------- ---------- ----------
CG017-5-33_T7              ACTGAGATCC GGCTGCTAAC AAAGCCCGAA AGGAAGCTGA GTTGGCTGCT

                           ....|....| ....|....| ....|....| ....|....| ....|....|
                                  710        720        730        740        750
Ref of CG017-5             ---------- ---------- ---------- ---------- ----------
CG017-5-33_T7              GCCACCGCTG AGCAATAACT AGCATAACCC CTTGGGGCCT CTAAACGGGT

                           ....|....| ....|....| ....|....| ....|....| ....|....|
                                  760        770        780        790        800
Ref of CG017-5             ---------- ---------- ---------- ---------- ----------
CG017-5-33_T7              CTTGAGGGGT TTTTTGCTGA AAGGAGGAAC TATATCCGGA TTGGCGAATG

                           ....|....| ....|....| ....|....| ....|....| ....|....|
                                  810        820        830        840        850
Ref of CG017-5             ---------- ---------- ---------- ---------- ----------
CG017-5-33_T7              GGACGCGCCC TGTAGCGGCG CATTAAGCGC GGCGGGTGTG GTGGTTACGC

                           ....|....| ....|....| ....|....| ....|....| ....|....|
                                  860        870        880        890        900
Ref of CG017-5             ---------- ---------- ---------- ---------- ----------
CG017-5-33_T7              GCAGCGTGAC CGCTACACTT GCCAGCGCCC TAGCGCCCGC TCCTTTCGCT

                           ....|....| ....|....| ....|....| ....|....| ....|....|
                                  910        920        930        940        950
Ref of CG017-5             ---------- ---------- ---------- ---------- ----------
CG017-5-33_T7              TTCTTCCCTT CCTTTCTCGC CACGTTCGCC GGCTTTCCCC GTCAAGCTCT
```
