# Supplementary material for: Rutin, A Natural Inhibitor of IGPD Protein, Partially Inhibits Biofilm Formation in Staphylococcus xylosus ATCC700404 in vitro and in vivo
Source: Front Pharmacol. 2021 Aug 11;12:728354. doi: 10.3389/fphar.2021.728354 (PMC8385535; doi:10.3389/fphar.2021.728354)
Supplement: Supplementary file 3 [file DataSheet4.zip › CG017-6 sequence alignment of añArg110 in pet30a IGPD .pdf]

```
                          ....|....| ....|....| ....|....| ....|....| ....|....|
                                 10         20         30         40         50
Ref of CG017-6            ---------- ---------- ---------- ---------- ------ATTT
CG017-6-33_T7             CCTCTAGAAT AATTTTGTTT AACTTTAAGA AGGAGATATA CATATGATTT

                          ....|....| ....|....| ....|....| ....|....| ....|....|
                                 60         70         80         90        100
Ref of CG017-6            ATCAAAAAAC ACGTAACACT GCTGAAACAC AACTATCTAT CTCACTTGCA
CG017-6-33_T7             ATCAAAAAAC ACGTAACACT GCTGAAACAC AATTATCTAT CTCACTTGCA

                          ....|....| ....|....| ....|....| ....|....| ....|....|
                                110        120        130        140        150
Ref of CG017-6            GATGACAATC GCCCAAGCAA AATCAACACT GGCGTGGGTT TTCTAGATCA
CG017-6-33_T7             GATGACAATC GCCCAAGCAA AATCAACACT GGCGTGGGTT TTCTAGATCA

                          ....|....| ....|....| ....|....| ....|....| ....|....|
                                160        170        180        190        200
Ref of CG017-6            TATGTTGACC CTCTTCACCT TTCATAGCAA CTTATCTATT ACTATCGAAG
CG017-6-33_T7             TATGTTGACC CTCTTCACCT TTCATAGCAA CTTATCTATT ACTATCGAAG

                          ....|....| ....|....| ....|....| ....|....| ....|....|
                                210        220        230        240        250
Ref of CG017-6            CAAATGGTGA TACAGAAGTA GATGATCACC ACGTCACAGA AGATATTGGT
CG017-6-33_T7             CAAATGGTGA TACAGAAGTA GACGATCACC ACGTCACAGA AGATATTGGT

                          ....|....| ....|....| ....|....| ....|....| ....|....|
                                260        270        280        290        300
Ref of CG017-6            ATTGTTTTAG GTCAATTGTT GTTAGAAATG ACTCGAGAAA GAAAATCCTT
CG017-6-33_T7             ATTGTTTTAG GTCAATTGTT GTTAGAAATG ACTCGAGAAA GAAAATCCTT

                          ....|....| ....|....| ....|....| ....|....| ....|....|
                                310        320        330        340        350
Ref of CG017-6            TCAACGTTAT GGCGTAAGTT ATATCCCTAT GGATGAAACA TTAGCACGTA
CG017-6-33_T7             TCAACGTTAT GGCGTAAGTT ATATCCCTAT GGATGAAACA TTAGCACGTA

                          ....|....| ....|....| ....|....| ....|....| ....|....|
                                360        370        380        390        400
Ref of CG017-6            CCGTCGTTGA TATTAGTGGA CGTCCTTTCC TTTCATTTAA TGCACATTTA
CG017-6-33_T7             CCGTCGTTGA TATTAGTGGA GCACCTTTCC TTTCATTTAA TGCGCATTTA

                          ....|....| ....|....| ....|....| ....|....| ....|....|
                                410        420        430        440        450
Ref of CG017-6            AGCCGTGAAA AGGTAGGCAC TTTTGATACG GAATTAGTAG AAGAATTCTT
CG017-6-33_T7             AGTCGTGAAA AGGTAGGCAC TTTTGATACG GAATTAGTAG AAGAATTCTT

                          ....|....| ....|....| ....|....| ....|....| ....|....|
                                460        470        480        490        500
Ref of CG017-6            CCGTGCATTA GTCATTAATG CACGCTTAAC AACGCATATT GATTTAATAC
CG017-6-33_T7             CCGTGCATTA GTCATTAATG CCCGCTTAAC AACGCATATT GATTTAATAC

                          ....|....| ....|....| ....|....| ....|....| ....|....|
                                510        520        530        540        550
Ref of CG017-6            GTGGTGGTAA TACCCACCAT GAAATAGAAG GAATCTTCAA ATCTTTTGCG
CG017-6-33_T7             GTGGTGGTAA TACTCACCAT GAAATAGAAG GAATCTTCAA ATCTTTTGCG
```

Email: gene-syn@comatebio.com TEL: (+86)431-85871624 Web: www.comatebio.com
Add: 3333 ShengBei St., Building E1, Changchun City, Jilin Province, 130000

```
                                   ....|....| ....|....| ....|....| ....|....| ....|....|
                                           560        570        580        590        600
Ref of CG017-6                     CGTGCACTTA AAGAATCTCT ATCAAGCAAT GACATCGACG GCACGCCGTC
CG017-6-33_T7                      CGTGCACTTA AAGAATCTCT ATCAAGCAAT GACATCAACG GCACGCCGTC

                                   ....|....| ....|....| ....|....| ....|....| ....|....|
                                           610        620        630        640        650
Ref of CG017-6                     ATCTAAGGGT GTGATAGAA- ---------- ---------- ----------
CG017-6-33_T7                      ATCTAAGGGT GTGATAGAAC TCGAGCACCA CCACCACCAC CACTGAGATC

                                   ....|....| ....|....| ....|....| ....|....| ....|....|
                                           660        670        680        690        700
Ref of CG017-6                     ---------- ---------- ---------- ---------- ----------
CG017-6-33_T7                      CGGCTGCTAA CAAAGCCCGA AAGGAAGCTG AGTTGGCTGC TGCCACCGCT

                                   ....|....| ....|....| ....|....| ....|....| ....|....|
                                           710        720        730        740        750
Ref of CG017-6                     ---------- ---------- ---------- ---------- ----------
CG017-6-33_T7                      GAGCAATAAC TAGCATAACC CCTTGGGGCC TCTAAACGGG TCTTGAGGGG

                                   ....|....| ....|....| ....|....| ....|....| ....|....|
                                           760        770        780        790        800
Ref of CG017-6                     ---------- ---------- ---------- ---------- ----------
CG017-6-33_T7                      TTTTTTGCTG AAAGGAGGAA CTATATCCGG ATTGGCGAAT GGGACGCGCC

                                   ....|....| ....|....| ....|....| ....|....| ....|....|
                                           810        820        830        840        850
Ref of CG017-6                     ---------- ---------- ---------- ---------- ----------
CG017-6-33_T7                      CTGTAGCGGC GCATTAAGCG CGGCGGGTGT GGTGGTTACG CGCAGCGTGA

                                   ....|....| ....|....| ....|....| ....|....| ....|....|
                                           860        870        880        890        900
Ref of CG017-6                     ---------- ---------- ---------- ---------- ----------
CG017-6-33_T7                      CCGCTACACT TGCCAGCGCC CTAGCGCCCG CTCCTTTCGC TTTCTTCCCT

                                   ....|....| ....|....| ....|....| ....|....| ....|....|
                                           910        920        930        940        950
Ref of CG017-6                     ---------- ---------- ---------- ---------- ----------
CG017-6-33_T7                      TCCTTTCTCG CCACGTTCGC CGGCTTTCCC CGTCAAGCTC TAAATCGGGG
```
