# Supplementary material for: Rutin, A Natural Inhibitor of IGPD Protein, Partially Inhibits Biofilm Formation in Staphylococcus xylosus ATCC700404 in vitro and in vivo
Source: Front Pharmacol. 2021 Aug 11;12:728354. doi: 10.3389/fphar.2021.728354 (PMC8385535; doi:10.3389/fphar.2021.728354)
Supplement: Supplementary file 3 [file DataSheet4.zip › CG017-7 sequence alignment of añHis159 in pet30a IGPD .pdf]

```
                       ....|....| ....|....| ....|....| ....|....| ....|....|
                              10         20         30         40         50
Ref of CG017-7         ---------- ---------- ---------- ---------- ------ATTT
CG017-7-41_T7          CCTCTAGAAT AATTTTGTTT AACTTTAAGA AGGAGATATA CATATGATTT

                       ....|....| ....|....| ....|....| ....|....| ....|....|
                              60         70         80         90        100
Ref of CG017-7         ATCAAAAAAC ACGTAACACT GCTGAAACAC AACTATCTAT CTCACTTGCA
CG017-7-41_T7          ATCAAAAAAC ACGTAACACT GCTGAAACAC AATTATCTAT CTCACTTGCA

                       ....|....| ....|....| ....|....| ....|....| ....|....|
                             110        120        130        140        150
Ref of CG017-7         GATGACAATC GCCCAAGCAA AATCAACACT GGCGTGGGTT TTCTAGATCA
CG017-7-41_T7          GATGACAATC GCCCAAGCAA AATCAACACT GGCGTGGGTT TTCTAGATCA

                       ....|....| ....|....| ....|....| ....|....| ....|....|
                             160        170        180        190        200
Ref of CG017-7         TATGTTGACC CTCTTCACCT TTCATAGCAA CTTATCTATT ACTATCGAAG
CG017-7-41_T7          TATGTTGACC CTCTTCACCT TTCATAGCAA CTTATCTATT ACTATCGAAG

                       ....|....| ....|....| ....|....| ....|....| ....|....|
                             210        220        230        240        250
Ref of CG017-7         CAAATGGTGA TACAGAAGTA GATGATCACC ACGTCACAGA AGATATTGGT
CG017-7-41_T7          CAAATGGTGA TACAGAAGTA GACGATCACC ACGTCACAGA AGATATTGGT

                       ....|....| ....|....| ....|....| ....|....| ....|....|
                             260        270        280        290        300
Ref of CG017-7         ATTGTTTTAG GTCAATTGTT GTTAGAAATG ACTCGAGAAA GAAAATCCTT
CG017-7-41_T7          ATTGTTTTAG GTCAATTGTT GTTAGAAATG ACTCGAGAAA GAAAATCCTT

                       ....|....| ....|....| ....|....| ....|....| ....|....|
                             310        320        330        340        350
Ref of CG017-7         TCAACGTTAT GGCGTAAGTT ATATCCCTAT GGATGAAACA TTAGCACGTA
CG017-7-41_T7          TCAACGTTAT GGCGTAAGTT ATATCCCTAT GGATGAAACA TTAGCACGTA

                       ....|....| ....|....| ....|....| ....|....| ....|....|
                             360        370        380        390        400
Ref of CG017-7         CCGTCGTTGA TATTAGTGGA CGTCCTTTCC TTTCATTTAA TGCACATTTA
CG017-7-41_T7          CCGTCGTTGA TATTAGTGGA CGTCCTTTCC TTTCATTTAA TGCGCATTTA

                       ....|....| ....|....| ....|....| ....|....| ....|....|
                             410        420        430        440        450
Ref of CG017-7         AGCCGTGAAA AGGTAGGCAC TTTTGATACG GAATTAGTAG AAGAATTCTT
CG017-7-41_T7          AGTCGTGAAA AGGTAGGCAC TTTTGATACG GAATTAGTAG AAGAATTCTT

                       ....|....| ....|....| ....|....| ....|....| ....|....|
                             460        470        480        490        500
Ref of CG017-7         CCGTGCATTA GTCATTAATG CACGCTTAAC AACGCATATT GATTTAATAC
CG017-7-41_T7          CCGTGCATTA GTCATTAATG CCCGCTTAAC AACGCATATT GATTTAATAC

                       ....|....| ....|....| ....|....| ....|....| ....|....|
                             510        520        530        540        550
Ref of CG017-7         GTGGTGGTAA TACCCACCAT GAAATAGAAG GAATCTTCAA ATCTTTTGCG
CG017-7-41_T7          GTGGTGGTAA TACCCACGCA GAAATAGAAG GAATCTTCAA ATCTTTTGCG
```

```
                              ....|....| ....|....| ....|....| ....|....| ....|....|
                                     560        570        580        590        600
Ref of CG017-7                CGTGCACTTA AAGAATCTCT ATCAAGCAAT GACATCGACG GCACGCCGTC
CG017-7-41_T7                 CGTGCACTTA AAGAATCTCT ATCAAGCAAT GACATCAACG GCACGCCGTC

                              ....|....| ....|....| ....|....| ....|....| ....|....|
                                     610        620        630        640        650
Ref of CG017-7                ATCTAAGGGT GTGATAGAA- ---------- ---------- ----------
CG017-7-41_T7                 ATCTAAGGGT GTGATAGAAC TCGAGCACCA CCACCACCAC CACTGAGATC

                              ....|....| ....|....| ....|....| ....|....| ....|....|
                                     660        670        680        690        700
Ref of CG017-7                ---------- ---------- ---------- ---------- ----------
CG017-7-41_T7                 CGGCTGCTAA CAAAGCCCGA AAGGAAGCTG AGTTGGCTGC TGCCACCGCT

                              ....|....| ....|....| ....|....| ....|....| ....|....|
                                     710        720        730        740        750
Ref of CG017-7                ---------- ---------- ---------- ---------- ----------
CG017-7-41_T7                 GAGCAATAAC TAGCATAACC CCTTGGGGCC TCTAAACGGG TCTTGAGGGG

                              ....|....| ....|....| ....|....| ....|....| ....|....|
                                     760        770        780        790        800
Ref of CG017-7                ---------- ---------- ---------- ---------- ----------
CG017-7-41_T7                 TTTTTTGCTG AAAGGAGGAA CTATATCCGG ATTGGCGAAT GGGACGCGCC

                              ....|....| ....|....| ....|....| ....|....| ....|....|
                                     810        820        830        840        850
Ref of CG017-7                ---------- ---------- ---------- ---------- ----------
CG017-7-41_T7                 CTGTAGCGGC GCATTAAGCG CGGCGGGTGT GGTGGTTACG CGCAGCGTGA

                              ....|....| ....|....| ....|....| ....|....| ....|....|
                                     860        870        880        890        900
Ref of CG017-7                ---------- ---------- ---------- ---------- ----------
CG017-7-41_T7                 CCGCTACACT TGCCAGCGCC CTAGCGCCCG CTCCTTTCGC TTTCTTCCCT

                              ....|....| ....|....| ....|....| ....|....| ....|....|
                                     910        920        930        940        950
Ref of CG017-7                ---------- ---------- ---------- ---------- ----------
CG017-7-41_T7                 TCCTTTCTCG CCACGTTCGC CGGCTTTCCC CGTCAAGCTC TAAATCGGGG
```
