# Supplementary material for: Rutin, A Natural Inhibitor of IGPD Protein, Partially Inhibits Biofilm Formation in Staphylococcus xylosus ATCC700404 in vitro and in vivo
Source: Front Pharmacol. 2021 Aug 11;12:728354. doi: 10.3389/fphar.2021.728354 (PMC8385535; doi:10.3389/fphar.2021.728354)
Supplement: Supplementary file 3 [file DataSheet4.zip › CG017-8 sequence alignment of añGlu162 in pet30a IGPD .pdf]

```
                        ....|....| ....|....| ....|....| ....|....| ....|....|
                                10         20         30         40         50
Ref of CG017-8          ---------- ---------- ---------- ---------- ----ATTTAT
CG017-8-97_T7           CTCTAGAATA TTTTGTTTAA CTTTAAGAAG GAGATATACA TATGATTTAT

                        ....|....| ....|....| ....|....| ....|....| ....|....|
                                60         70         80         90        100
Ref of CG017-8          CAAAAAACAC GTAACACTGC TGAAACACAA CTATCTATCT CACTTGCAGA
CG017-8-97_T7           CAAAAAACAC GTAACACTGC TGAAACACAA TTATCTATCT CACTTGCAGA

                        ....|....| ....|....| ....|....| ....|....| ....|....|
                               110        120        130        140        150
Ref of CG017-8          TGACAATCGC CCAAGCAAAA TCAACACTGG CGTGGGTTTT CTAGATCATA
CG017-8-97_T7           TGACAATCGC CCAAGCAAAA TCAACACTGG CGTGGGTTTT CTAGATCATA

                        ....|....| ....|....| ....|....| ....|....| ....|....|
                               160        170        180        190        200
Ref of CG017-8          TGTTGACCCT CTTCACCTTT CATAGCAACT TATCTATTAC TATCGAAGCA
CG017-8-97_T7           TGTTGACCCT CTTCACCTTT CATAGCAACT TATCTATTAC TATCGAAGCA

                        ....|....| ....|....| ....|....| ....|....| ....|....|
                               210        220        230        240        250
Ref of CG017-8          AATGGTGATA CAGAAGTAGA TGATCACCAC GTCACAGAAG ATATTGGTAT
CG017-8-97_T7           AATGGTGATA CAGAAGTAGA CGATCACCAC GTCACAGAAG ATATTGGTAT

                        ....|....| ....|....| ....|....| ....|....| ....|....|
                               260        270        280        290        300
Ref of CG017-8          TGTTTTAGGT CAATTGTTGT TAGAAATGAC TCGAGAAAGA AAATCCTTTC
CG017-8-97_T7           TGTTTTAGGT CAATTGTTGT TAGAAATGAC TCGAGAAAGA AAATCCTTTC

                        ....|....| ....|....| ....|....| ....|....| ....|....|
                               310        320        330        340        350
Ref of CG017-8          AACGTTATGG CGTAAGTTAT ATCCCTATGG ATGAAACATT AGCACGTACC
CG017-8-97_T7           AACGTTATGG CGTAAGTTAT ATCCCTATGG ATGAAACATT AGCACGTACC

                        ....|....| ....|....| ....|....| ....|....| ....|....|
                               360        370        380        390        400
Ref of CG017-8          GTCGTTGATA TTAGTGGACG TCCTTTCCTT TCATTTAATG CACATTTAAG
CG017-8-97_T7           GTCGTTGATA TTAGTGGACG TCCTTTCCTT TCATTTAATG CGCATTTAAG

                        ....|....| ....|....| ....|....| ....|....| ....|....|
                               410        420        430        440        450
Ref of CG017-8          CCGTGAAAAG GTAGGCACTT TTGATACGGA ATTAGTAGAA GAATTCTTCC
CG017-8-97_T7           TCGTGAAAAG GTAGGCACTT TTGATACGGA ATTAGTAGAA GAATTCTTCC

                        ....|....| ....|....| ....|....| ....|....| ....|....|
                               460        470        480        490        500
Ref of CG017-8          GTGCATTAGT CATTAATGCA CGCTTAACAA CGCATATTGA TTTAATACGT
CG017-8-97_T7           GTGCATTAGT CATTAATGCC CGCTTAACAA CGCATATTGA TTTAATACGT

                        ....|....| ....|....| ....|....| ....|....| ....|....|
                               510        520        530        540        550
Ref of CG017-8          GGTGGTAATA CCCACCATGA AATAGAAGGA ATCTTCAAAT CTTTTGCGCG
CG017-8-97_T7           GGTGGTAATA CCCACCATGA AATAGCAGGA ATCTTCAAAT CTTTTGCGCG
```

```
                        ....|....| ....|....| ....|....| ....|....| ....|....|
                               560        570        580        590        600
Ref of CG017-8          TGCACTTAAA GAATCTCTAT CAAGCAATGA CATCGACGGC ACGCCGTCAT
CG017-8-97_T7           TGCACTTAAA GAATCTCTAT CAAGCAATGA CATCAACGGC ACGCCGTCAT

                        ....|....| ....|....| ....|....| ....|....| ....|....|
                               610        620        630        640        650
Ref of CG017-8          CTAAGGGTGT GATAGAA--- ---------- ---------- ----------
CG017-8-97_T7           CTAAGGGTGT GATAGAACTC GAGCACCACC ACCACCACCA CTGAGATCCG

                        ....|....| ....|....| ....|....| ....|....| ....|....|
                               660        670        680        690        700
Ref of CG017-8          ---------- ---------- ---------- ---------- ----------
CG017-8-97_T7           GCTGCTAACA AAGCCCGAAA GGAAGCTGAG TTGGCTGCTG CCACCGCTGA

                        ....|....| ....|....| ....|....| ....|....| ....|....|
                               710        720        730        740        750
Ref of CG017-8          ---------- ---------- ---------- ---------- ----------
CG017-8-97_T7           GCAATAACTA GCATAACCCC TTGGGGCCTC TAAACGGGTC TTGAGGGGTT

                        ....|....| ....|....| ....|....| ....|....| ....|....|
                               760        770        780        790        800
Ref of CG017-8          ---------- ---------- ---------- ---------- ----------
CG017-8-97_T7           TTTTGCTGAA AGGAGGAACT ATATCCGGAT TGGCGAATGG GACGCGCCCT

                        ....|....| ....|....| ....|....| ....|....| ....|....|
                               810        820        830        840        850
Ref of CG017-8          ---------- ---------- ---------- ---------- ----------
CG017-8-97_T7           GTAGCGGCGC ATTAAGCGCG GCGGGTGTGG TGGTTACGCG CAGCGTGACC

                        ....|....| ....|....| ....|....| ....|....| ....|....|
                               860        870        880        890        900
Ref of CG017-8          ---------- ---------- ---------- ---------- ----------
CG017-8-97_T7           GCTACACTTG CCAGCGCCCT AGCGCCCGCT CCTTTCGCTT TCTTCCCTTC

                        ....|....| ....|....| ....|....| ....|....| ....|....|
                               910        920        930        940        950
Ref of CG017-8          ---------- ---------- ---------- ---------- ----------
CG017-8-97_T7           CTTTCTCGCC ACGTTCGCCG GCTTTCCCCG TCAAGCTCTA AATCGGGGGC
```
