# Supplementary material for: Rutin, A Natural Inhibitor of IGPD Protein, Partially Inhibits Biofilm Formation in Staphylococcus xylosus ATCC700404 in vitro and in vivo
Source: Front Pharmacol. 2021 Aug 11;12:728354. doi: 10.3389/fphar.2021.728354 (PMC8385535; doi:10.3389/fphar.2021.728354)
Supplement: Supplementary file 3 [file DataSheet4.zip › CG017-9 sequence alignment of añLys166 in pet30a IGPD .pdf]

```
                         ....|....| ....|....| ....|....| ....|....| ....|....|
                                 10         20         30         40         50
Ref of CG017-9           ---------- ---------- ---------- ---------- ----------
CG017-9-106_T7           GTAGACGGTC ATTGCTTCTA GAATAATTTT GTTTAACTTT AAGAAGGAGA

                         ....|....| ....|....| ....|....| ....|....| ....|....|
                                 60         70         80         90        100
Ref of CG017-9           ---------- ATTTATCAAA AAACACGTAA CACTGCTGAA ACACAACTAT
CG017-9-106_T7           TATACATATG ATTTATCAAA AAACACGTAA CACTGCTGAA ACACAATTAT

                         ....|....| ....|....| ....|....| ....|....| ....|....|
                                110        120        130        140        150
Ref of CG017-9           CTATCTCACT TGCAGATGAC AATCGCCCAA GCAAAATCAA CACTGGCGTG
CG017-9-106_T7           CTATCTCACT TGCAGATGAC AATCGCCCAA GCAAAATCAA CACTGGCGTG

                         ....|....| ....|....| ....|....| ....|....| ....|....|
                                160        170        180        190        200
Ref of CG017-9           GGTTTTCTAG ATCATATGTT GACCCTCTTC ACCTTTCATA GCAACTTATC
CG017-9-106_T7           GGTTTTCTAG ATCATATGTT GACCCTCTTC ACCTTTCATA GCAACTTATC

                         ....|....| ....|....| ....|....| ....|....| ....|....|
                                210        220        230        240        250
Ref of CG017-9           TATTACTATC GAAGCAAATG GTGATACAGA AGTAGATGAT CACCACGTCA
CG017-9-106_T7           TATTACTATC GAAGCAAATG GTGATACAGA AGTAGACGAT CACCACGTCA

                         ....|....| ....|....| ....|....| ....|....| ....|....|
                                260        270        280        290        300
Ref of CG017-9           CAGAAGATAT TGGTATTGTT TTAGGTCAAT TGTTGTTAGA AATGACTCGA
CG017-9-106_T7           CAGAAGATAT TGGTATTGTT TTAGGTCAAT TGTTGTTAGA AATGACTCGA

                         ....|....| ....|....| ....|....| ....|....| ....|....|
                                310        320        330        340        350
Ref of CG017-9           GAAAGAAAAT CCTTTCAACG TTATGGCGTA AGTTATATCC CTATGGATGA
CG017-9-106_T7           GAAAGAAAAT CCTTTCAACG TTATGGCGTA AGTTATATCC CTATGGATGA

                         ....|....| ....|....| ....|....| ....|....| ....|....|
                                360        370        380        390        400
Ref of CG017-9           AACATTAGCA CGTACCGTCG TTGATATTAG TGGACGTCCT TTCCTTTCAT
CG017-9-106_T7           AACATTAGCA CGTACCGTCG TTGATATTAG TGGACGTCCT TTCCTTTCAT

                         ....|....| ....|....| ....|....| ....|....| ....|....|
                                410        420        430        440        450
Ref of CG017-9           TTAATGCACA TTTAAGCCGT GAAAAGGTAG GCACTTTTGA TACGGAATTA
CG017-9-106_T7           TTAATGCGCA TTTAAGTCGT GAAAAGGTAG GCACTTTTGA TACGGAATTA

                         ....|....| ....|....| ....|....| ....|....| ....|....|
                                460        470        480        490        500
Ref of CG017-9           GTAGAAGAAT TCTTCCGTGC ATTAGTCATT AATGCACGCT TAACAACGCA
CG017-9-106_T7           GTAGAAGAAT TCTTCCGTGC ATTAGTCATT AATGCCCGCT TAACAACGCA

                         ....|....| ....|....| ....|....| ....|....| ....|....|
                                510        520        530        540        550
Ref of CG017-9           TATTGATTTA ATACGTGGTG GTAATACCCA CCATGAAATA GAAGGAATCT
CG017-9-106_T7           TATTGATTTA ATACGTGGTG GTAATACTCA CCATGAAATA GAAGGAATCT
```

```
                       ....|....| ....|....| ....|....| ....|....| ....|....|
                              560        570        580        590        600
Ref of CG017-9         TCAAATCTTT TGCGCGTGCA CTTAAAGAAT CTCTATCAAG CAATGACATC
CG017-9-106_T7         TCGCATCTTT TGCGCGTGCA CTTAAAGAAT CTCTATCAAG CAATGACATC

                       ....|....| ....|....| ....|....| ....|....| ....|....|
                              610        620        630        640        650
Ref of CG017-9         GACGGCACGC CGTCATCTAA GGGTGTGATA GAA------- ----------
CG017-9-106_T7         AACGGCACGC CGTCATCTAA GGGTGTGATA GAACTCGAGC ACCACCACCA

                       ....|....| ....|....| ....|....| ....|....| ....|....|
                              660        670        680        690        700
Ref of CG017-9         ---------- ---------- ---------- ---------- ----------
CG017-9-106_T7         CCACCACTGA GATCCGGCTG CTAACAAAGC CCGAAAGGAA GCTGAGTTGG

                       ....|....| ....|....| ....|....| ....|....| ....|....|
                              710        720        730        740        750
Ref of CG017-9         ---------- ---------- ---------- ---------- ----------
CG017-9-106_T7         CTGCTGCCAC CGCTGAGCAA TAACTAGCAT AACCCCTTGG GGCCTCTAAA

                       ....|....| ....|....| ....|....| ....|....| ....|....|
                              760        770        780        790        800
Ref of CG017-9         ---------- ---------- ---------- ---------- ----------
CG017-9-106_T7         CGGGTCTTGA GGGGTTTTTT GCTGAAAGGA GGAACTATAT CCGGATTGGC

                       ....|....| ....|....| ....|....| ....|....| ....|....|
                              810        820        830        840        850
Ref of CG017-9         ---------- ---------- ---------- ---------- ----------
CG017-9-106_T7         GAATGGGACG CGCCCTGTAG CGGCGCATTA AGCGCGGCGG GTGTGGTGGT

                       ....|....| ....|....| ....|....| ....|....| ....|....|
                              860        870        880        890        900
Ref of CG017-9         ---------- ---------- ---------- ---------- ----------
CG017-9-106_T7         TACGCGCAGC GTGACCGCTA CACTTGCCAG CGCCCTAGCG CCCGCTCCTT
```

Email: gene-syn@comatebio.com TEL: (+86)431-85871624 Web: www.comatebio.com
Add: 3333 ShengBei St., Building E1, Changchun City, Jilin Province, 130000
