# Supplementary material for: Rutin, A Natural Inhibitor of IGPD Protein, Partially Inhibits Biofilm Formation in Staphylococcus xylosus ATCC700404 in vitro and in vivo
Source: Front Pharmacol. 2021 Aug 11;12:728354. doi: 10.3389/fphar.2021.728354 (PMC8385535; doi:10.3389/fphar.2021.728354)
Supplement: Supplementary file 4 [file DataSheet1.pdf]

S1 Fig: A brief flow chart of the BLI experiment.

Buffer: PBS buffer of pH 6.5; His-tagged: His-tag IGPD recombinant protein, including original protein and amino acid site-directed mutant protein; Buffer 2: The buffer 2 used was PBS containing 0.1% bovine serum albumin and 0.02% Tween-20, pH 6.5; Rutin: Rutin is dissolved in buffer 2.

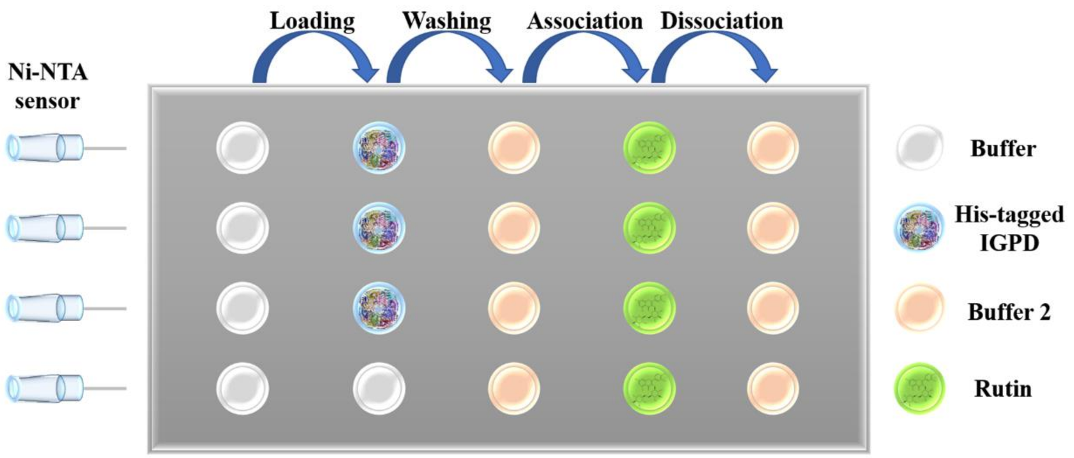

S2 Fig: The molecular dynamics (MD) simulation of the IGPD/rutin complex.

The root-mean-square deviation (RMSD) calculated for the backbone atoms of the protein during MD simulation.

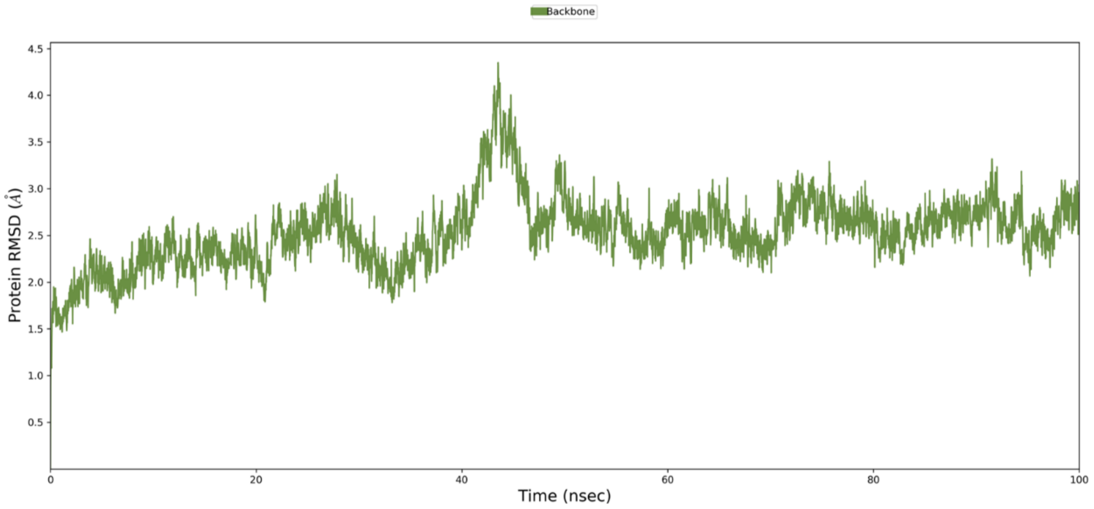

S3 Fig: A representative sequencing chromatogram of nine IGPD mutant proteins. The IGPD sequence of wild type was used as control.

A: Sequencing results of pET30a-Ala7. **B:** Sequencing results of pET30a-Ala62. **C:** Sequencing results of pET30a-Ala63. **D:** Sequencing results of pET30a-Ala66. **E:** Sequencing results of pET30a-Ala97. **F:** Sequencing results of pET30a-Ala110. **G:** Sequencing results of pET30a-Ala159. **H:** Sequencing results of pET30a-Ala162. **I:** Sequencing results of pET30a-Ala166.

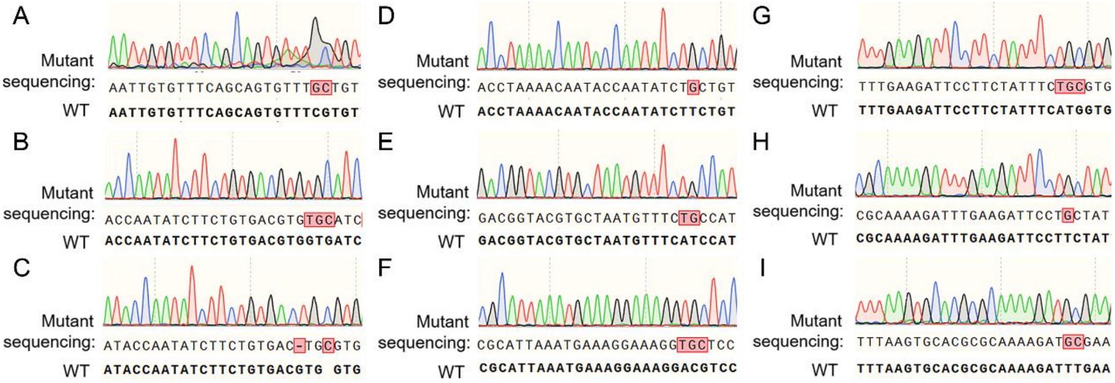

S4 Fig: SDS-PAGE gel analysis of IGPD and its mutant proteins expression as induced by the presence or absence IPTG.

Lane 1: Protein molecular mass marker; lane 2: The lysates of BL21 (DE3) cells containing pET30a-IGPD without IPTG; Lane 3: The lysates of BL21 (DE3) cells containing pET30a-IGPD with IPTG; Lane 4: The supernatants of BL21 (DE3) cells containing pET30a-IGPD with IPTG; Lane 5: The precipitates of BL21 (DE3) cells containing pET30a-IGPD with IPTG. **A:** IGPD. **B:** IGPD ΔArg7. **C:** IGPD ΔHis62. **D:** IGPD ΔHis63. **E:** IGPD ΔGlu66. **F:** IGPD ΔAsp97. **G:** IGPD ΔArg110. **H:** IGPD ΔHis159. **I:** IGPD ΔGlu162. **J:** IGPD ΔLys166.

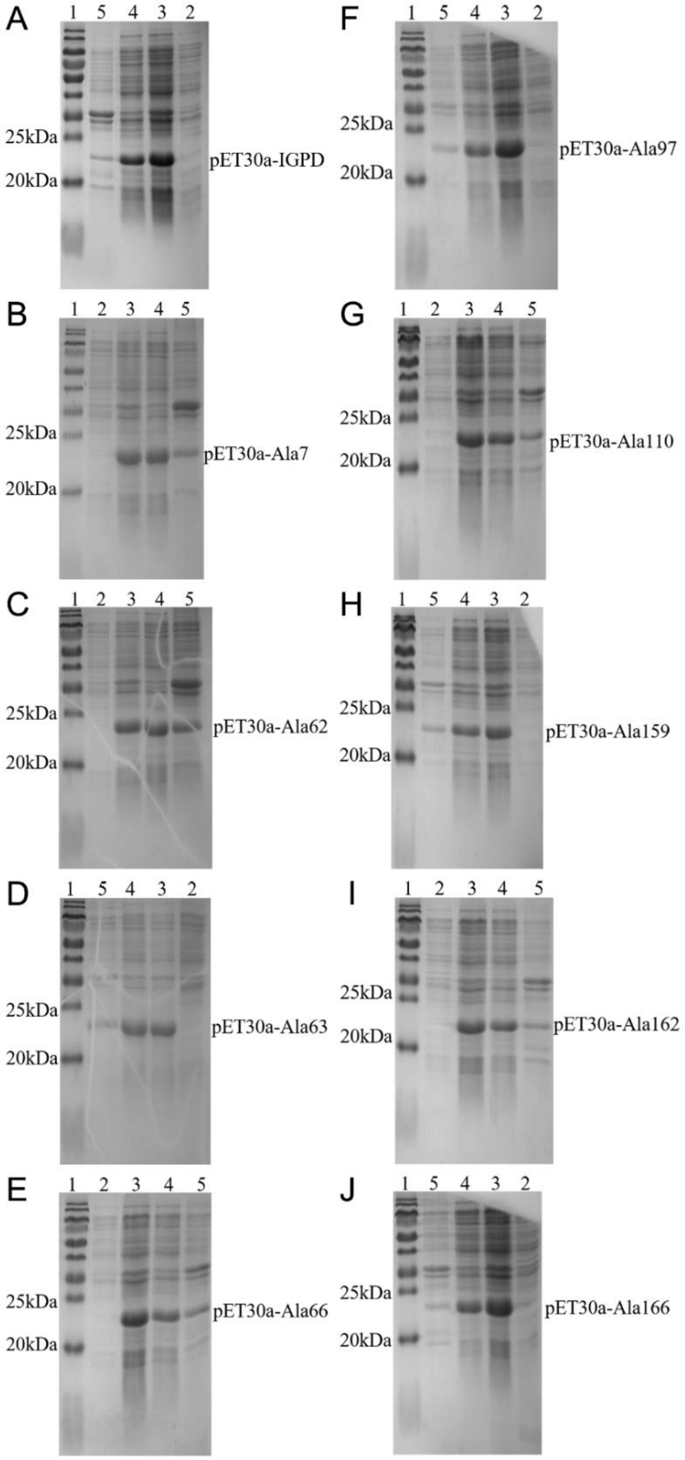

S5 Fig: AKTA purified protein absorption curve of IGPD and its mutant proteins.

A: IGPD. **B:** IGPD ΔArg7. **C:** IGPD ΔHis62. **D:** IGPD ΔHis63. **E:** IGPD ΔGlu66. **F:** IGPD ΔAsp97. **G:** IGPD ΔArg110. **H:** IGPD ΔHis159. **I:** IGPD ΔGlu162. **J:** IGPD ΔLys166.

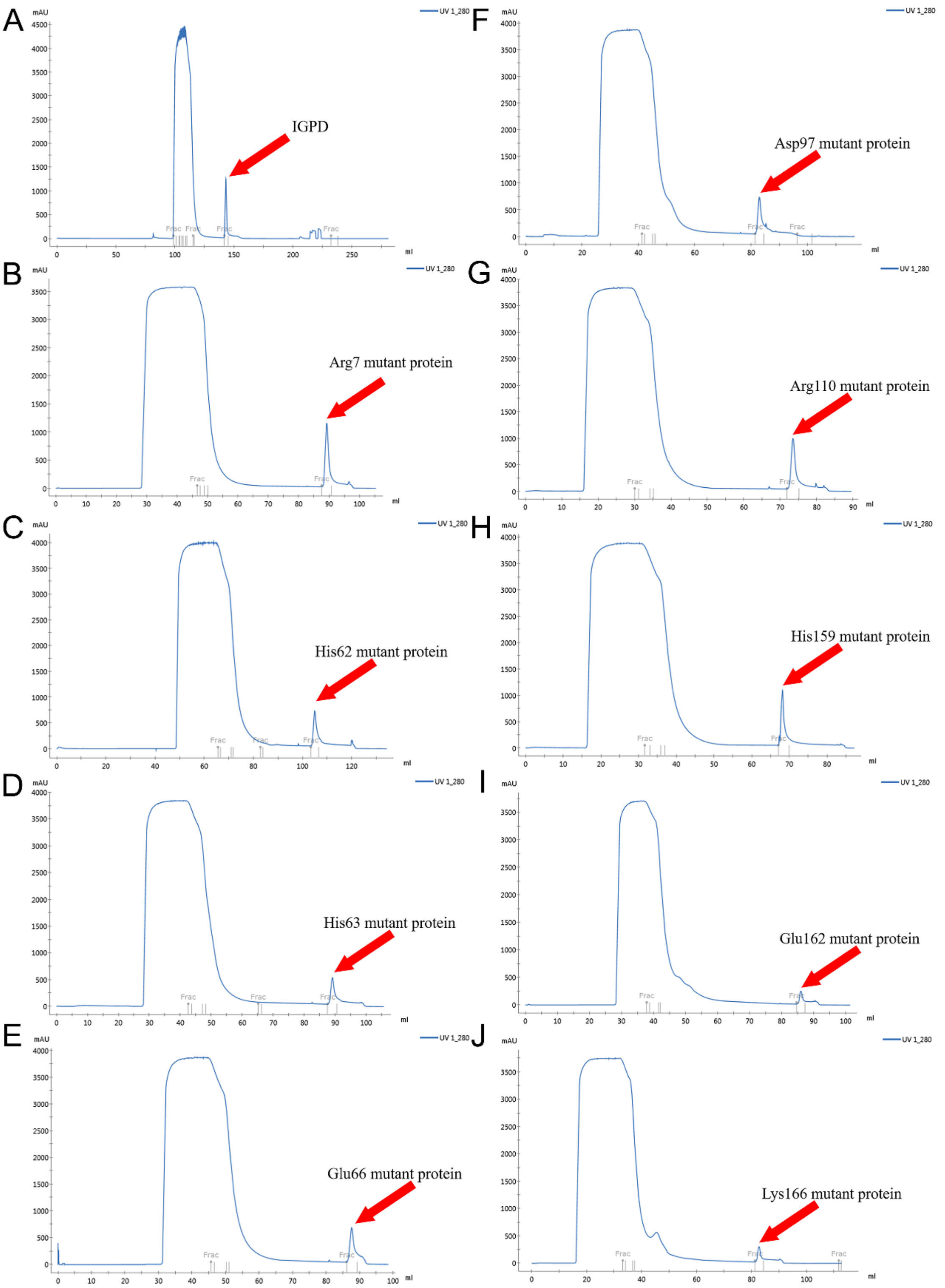

S6 Fig: Interaction between rutin and mutant protein.

A: Interaction between IGPD ΔArg7 and rutin. **B:** Interaction between IGPD ΔHis63 and rutin. **C:** Interaction between IGPD ΔGlu66 and rutin. **D:** Interaction between IGPD ΔAsp97 and rutin. **E:** Interaction between IGPD ΔArg110 and rutin. **F:** Interaction between IGPD ΔHis159 and rutin. **G:** Interaction between IGPD ΔGlu162 and rutin. **H:** Interaction between IGPD ΔLys166 and rutin.

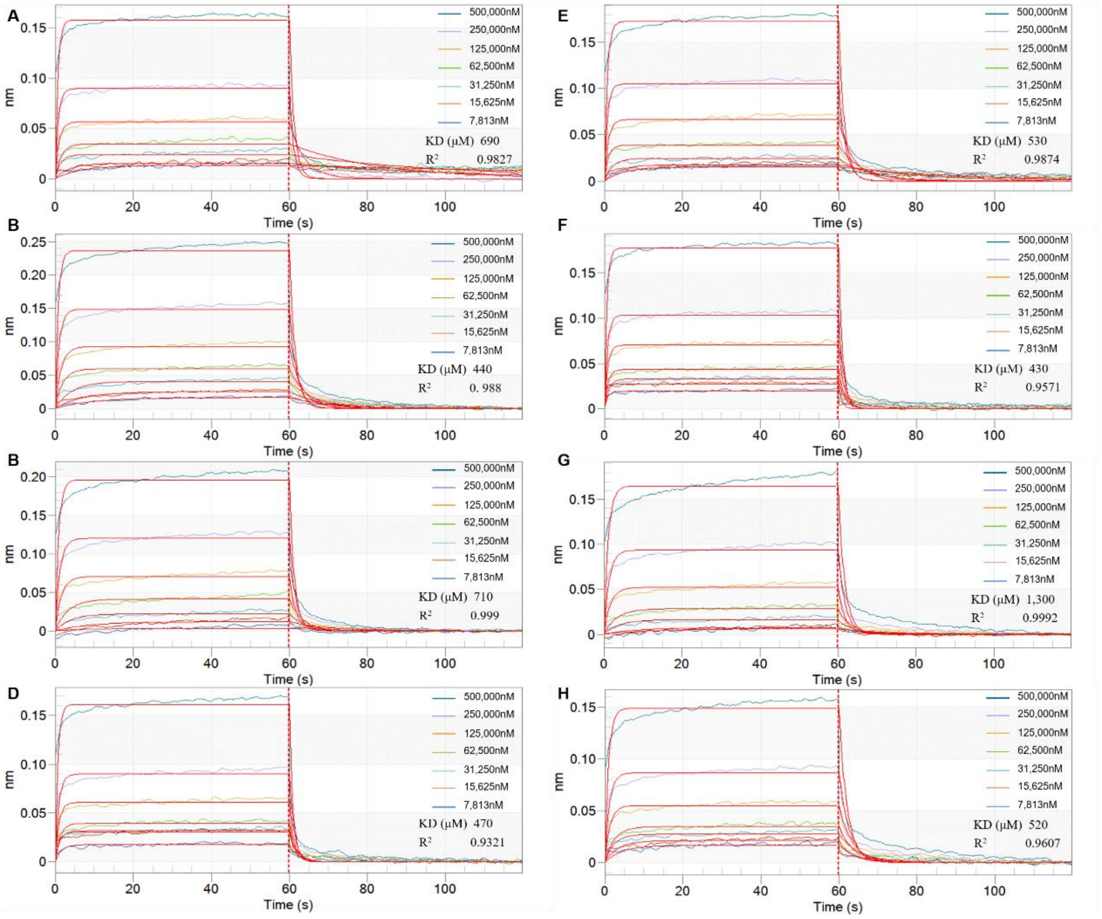

Original images for blots

Fig. 3_B_raw_images

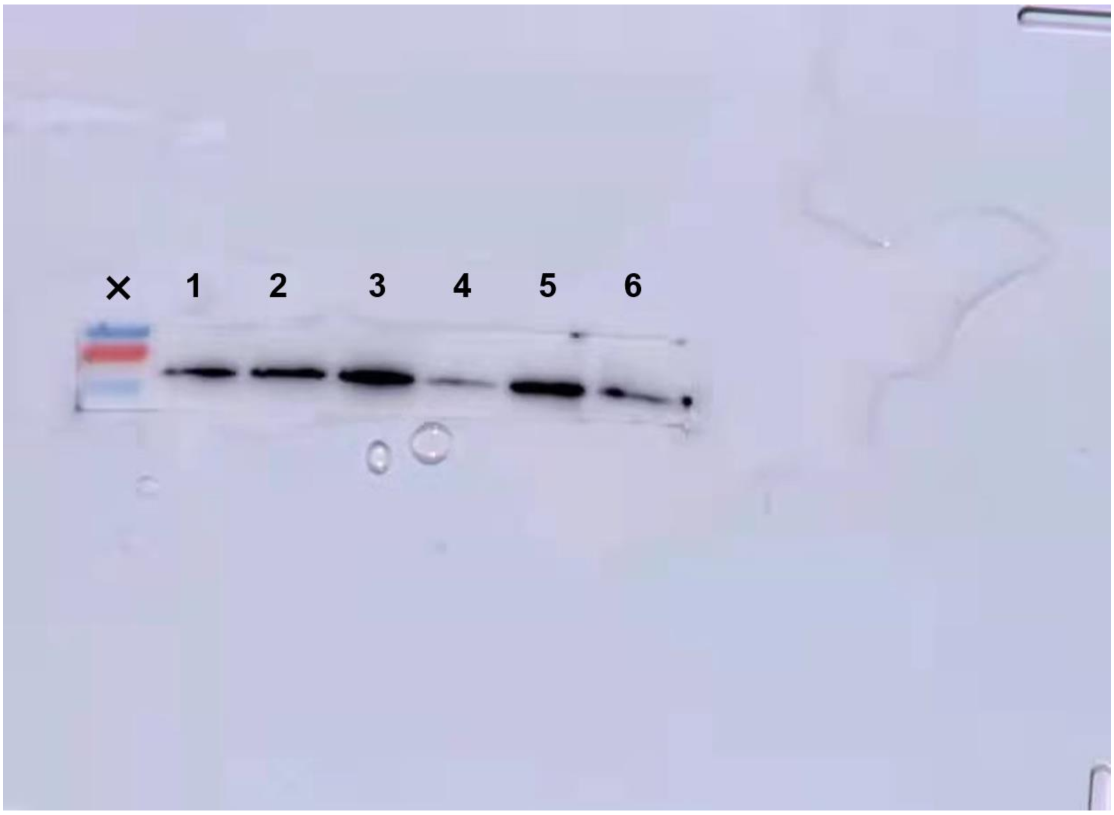

The effect of rutin on the expression of IGPD protein in *S. xylosus* at 6, 12, and 24 h, respectively. Line 1: 6 h without rutin; Line 2: 6 h with rutin; Line 3: 12 h without rutin; Line 4: 12 h with rutin; Line 5: 24 h without rutin; Line 6: 24 h with rutin.
